# Toward a universal framework for evaluating transport resistances and driving forces in membrane-based desalination processes

**DOI:** 10.1126/sciadv.ade0413

**Published:** 2023-01-04

**Authors:** Kian P. Lopez, Ruoyu Wang, Elizabeth A. Hjelvik, Shihong Lin, Anthony P. Straub

**Affiliations:** ^1^ Department of Civil, Environmental and Architectural Engineering, University of Colorado Boulder, Boulder, CO 80309-0428, USA.; ^2^ Department of Civil and Environmental Engineering, Vanderbilt University, Nashville, TN 37235-1831, USA.; ^3^ Materials Science and Engineering Program, University of Colorado Boulder, Boulder, CO 80309-0428, USA.

## Abstract

Desalination technologies using salt-rejecting membranes are a highly efficient tool to provide fresh water and augment existing water supplies. In recent years, numerous studies have worked to advance a variety of membrane processes with different membrane types and driving forces, but direct quantitative comparisons of these different technologies have led to confusing and contradictory conclusions in the literature. In this Review, we critically assess different membrane-based desalination technologies and provide a universal framework for comparing various driving forces and membrane types. To accomplish this, we first quantify the thermodynamic driving forces resulting from pressure, concentration, and temperature gradients. We then examine the resistances experienced by water molecules as they traverse liquid- and air-filled membranes. Last, we quantify water fluxes in each process for differing desalination scenarios. We conclude by synthesizing results from the literature and our quantitative analyses to compare desalination processes, identifying specific scenarios where each process has fundamental advantages.

## Teaser

Advantages of various membrane-based desalination processes are compared by analyzing driving forces and transport principles.

## INTRODUCTION

Climate change, population growth, and industrialization are stressing global water supplies (*1*). Addressing the challenge of water scarcity motivates the development of desalination technologies that can augment existing freshwater supplies resulting from the hydrogeologic water cycle (*2*). Membrane-based processes have emerged as some of the most widely considered technologies for desalination owing to their high energy efficiency, ease of implementation, and consistently high product water quality. Currently, membrane-based processes are the premier technologies used in brackish water and seawater desalination, and there is growing interest in using membrane-based systems for treating other source waters (*3*). The success of membrane-based systems has motivated the improvement of material and process design to further enhance energy efficiency, product water quality, and environmental sustainability.

Membrane-based processes rely on (i) a membrane that selectively discriminates between water and dissolved salts and (ii) a driving force for mass transfer. The salt-rejecting membranes in current membrane-based desalination processes are typically made of thin films of polymeric materials that facilitate preferential transport of water molecules as compared to ions and other solutes (*4*). This selective transport is the result of angstrom-scale free volume elements within the polymer materials that sterically, and sometimes electrostatically, inhibit the transport of hydrated ions and larger solutes (*5*). Common dense polymers used in salt-rejecting membranes include cross-linked polyamide and cellulose acetate. Other emerging membrane materials that rely on steric and electrostatic rejection mechanisms are being increasingly considered, including nanopores, graphene-based films, and artificial water channels (*6*).

Although dense polymer membranes are by far the most widely implemented in desalination processes, air-filled membranes used in membrane-based distillation processes have gained growing interest. These membranes use a hydrophobic porous matrix to trap air within their pores, so that water transport through these membranes only occurs in the gas phase, i.e., water vapor can travel through the membrane, but all nonvolatile solutes are rejected (*7*–*10*). Typically, air-filled membranes are made of hydrophobic polymers with materials such as polytetrafluoroethylene, polyvinylidene fluoride, and polypropylene. Air-filled membranes are being considered as potential substitutes for conventional dense polymer membranes in certain applications due to their higher rejection of nonvolatile solutes, improved resistance to oxidative chemicals, and sometimes higher transport rates (*11*–*15*).

For processes using salt-rejecting membranes, the driving force for transport is based on differences in either hydraulic pressure, concentration, or temperature across the membrane. For example, a hydraulic pressure is used in reverse osmosis (RO)—a mature technology that has already been implemented at a large scale for desalination of seawater and brackish water. Concentration differences are used in forward osmosis (FO) and osmotic distillation (OD) processes, which have been widely investigated in research laboratories and, in the case of FO, piloted for the desalination of high-salinity brines (*16*–*18*). Temperature differences are used in membrane distillation (MD) systems, which have experienced growing interest for the treatment of high-salinity brines (*7*, *19*).

Despite processes using various membrane types and driving forces all working toward the same goal of water desalination, direct comparison of different membrane-based desalination systems is difficult and has led to confusing, often contradictory, results in the literature. For example, dense polymer membranes have shown fluxes orders of magnitude higher than air-filled membranes in pressure- and concentration-driven systems, but certain studies have shown conflicting ultrahigh fluxes for air-filled membranes (*20*–*22*). In temperature-driven processes, MD systems using air-filled membranes have shown high fluxes even when treating high-salinity brines, while dense polymer membranes show low and sometimes negative flux values (*19*, *23*). The challenge of relating unique processes invites the implementation of a broad but unified framework that can allow for the direct comparison of a variety of fundamentally different membrane-based desalination systems driven by hydraulic pressure, concentration, and temperature differences. This framework is, in theory, possible because all processes rely on quantifiable thermodynamic driving forces and transport resistances. Such a framework would allow for an improved understanding of scenarios where each process can offer fundamental advantages.

In this analysis, we critically assess different membrane-based desalination technologies and provide a universal framework for comparing various driving forces and membrane classes. To accomplish this, we first quantify the thermodynamic driving forces resulting from pressure, concentration, and temperature gradients across salt-rejecting membranes. We then examine transport resistances that occur as molecules and ions travel through common desalination membranes. Building on these analyses, we synthesize results from the literature to quantitatively and qualitatively compare desalination processes, identifying specific scenarios where each process has benefits. Overall, this review will summarize fundamental advantages of different desalination methods and applications where each process can be most efficiently implemented.

## WHAT ARE THE DRIVING FORCES FROM PRESSURE, CONCENTRATION, AND TEMPERATURE GRADIENTS?

**Fig. 1.**
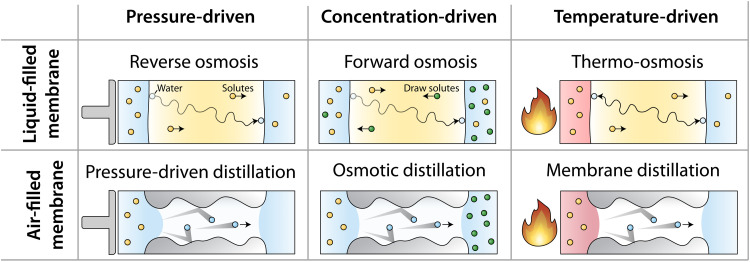
Summary of desalination processes driven by hydraulic pressure, concentration, and temperature gradients using either liquid- or air-filled membranes.

The six membrane desalination processes that occur from driving forces of pressure, concentration, and temperature in liquid- and air-filled membranes are illustrated in Fig. 1. For the broad framework of this analysis, “liquid-filled” is used to describe membranes in which water transport occurs in the liquid (or sorbed) phase (e.g., dense polymers, graphene, and artificial water channels), and “air-filled” is used to describe distillation membranes in which water transport occurs in the vapor phase. Pressure-driven desalination uses liquid-filled membranes in RO and air-filled membranes in pressure-driven distillation (PD). In both RO and PD, pressure applied to the feed side of the membrane generates a difference in chemical potential between the feed and permeate (*24*–*26*). The difference in chemical potential across the membrane causes water to pass through the membrane. The applied pressure must be greater than the osmotic pressure of the feed solution for desalination to occur. Concentration-driven desalination takes place in liquid- and air-filled membranes as FO and OD, respectively. Both FO and OD use draw solutions (i.e., a solution with a high concentration of solutes and thus a high osmotic pressure) to create a difference in osmotic pressure across the membrane (*16*, *27*). This gradient in osmotic pressure between the feed and draw results in a difference in chemical potential, causing water to traverse the membrane. Temperature-driven desalination in air-filled membranes is used in MD where a difference of the temperatures at the liquid-air interfaces creates a chemical potential gradient manifested as the partial vapor pressure difference generated between the feed and permeate sides of the membrane (*7*, *28*). When a temperature difference occurs across a dense polymer membrane, thermal migration occurs in a nonequilibrium exchange of mass and heat flux that causes water permeation in a process called thermo-osmosis (TO) (*23*, *29*–*31*). It should be noted that a driving force for desalination can also be generated from an electric potential (i.e., electrodialysis); however, electric potentials are used to drive ion transport rather than water transport (*32*). To allow for logical comparison between processes, this analysis will only focus on salt-rejecting membranes with water transport driven by gradients in pressure, concentration, and temperature.

### Defining thermodynamic driving forces

The driving forces for water permeation through liquid- and air-filled membranes are gradients in pressure, concentration, and temperature (*33*, *34*). These gradients can be quantitatively related to mass and heat transport in membrane systems using nonequilibrium thermodynamics, where entropy production in the membrane is expressed as a function of gradients in both chemical potential and temperature (*23*, *29*). Transport of heat and mass can be related through the coupling of force-flux relationships. This association allows for mass flux to be expressed in terms of heat flux and vice versa (*35*). Further explanation of how entropy production is used to relate pressure-, concentration-, and temperature-driven membrane systems can be found in the Supplementary Materials.

Thermodynamic analysis of the driving forces for transmembrane permeation in both liquid- and air-filled membranes facilitates comparisons between seemingly different processes. Taking entropy production of single-component heat and mass transport where chemical potential is a function of pressure and concentration and heat flux is a function of temperature allows for the derivation of a universal equation relating driving forces to mass flux across liquid- and air-filled membranes

$$J_{w}=A\left(\Delta P_{h}-\Delta \pi +\frac{Q^{*}}{V_{w}T}\Delta T\right)$$ (1)

where *J*_w_ is the volumetric water flux, *A* is a proportionality factor here referred to as the water permeability coefficient, ∆*P*_h_ is the difference in hydraulic pressure between the feed and permeate, ∆π is the difference in osmotic pressure between the feed and permeate, Q^*^ is the heat transferred per mole of fluid permeating across the membrane, *V*_w_ is the molar volume of liquid water, *T* is the average temperature across the membrane, and ∆*T* is the difference in temperature between the feed and permeate (*23*, *29*). Mass and heat flux can be related through the Onsager relations allowing for Eq. 1 to relate water flux gradients in pressure, concentration, and temperature for both liquid- and air-filled membranes (*35*). Further explanation of the derivation of Eq. 1 and correlations between hydraulic, osmotic, and vapor pressure can be found in the Supplementary Materials.

Defining the individual terms in Eq. 1 allows for a direct comparison of the driving forces resulting from concentration, pressure, and temperature differences across a salt-rejecting membrane. The applied pressure term, Δ*P*_h_, is straightforward because an increase in hydraulic pressure on the feed side results in a corresponding increase in chemical potential (*24*). The chemical potential gradient formed by an applied pressure is the same for both liquid- and air-filled membranes, meaning that both classes of membranes under applied pressures have equivalent driving forces when boundary layer effects are not considered. While the effect of an applied pressure on liquid-filled membranes has been thoroughly studied in RO systems, experimental validation in air-filled membranes is a more recent development (*20*, *25*, *36*).

The driving force from a concentration difference is manifested in the osmotic pressure difference, ∆π, term of Eq. 1 that quantifies the impact of concentration on the chemical potential gradient across the membrane (*24*, *37*). The osmotic pressure can be determined using the Van’t Hoff equation: $\pi =\frac{R_{g}TvM}{V_{w}}m\Phi ,$ where π is the osmotic pressure, *M* is the molar mass of water, Φ is the osmotic coefficient accounting for nonideal interactions, *v* is the Van’t Hoff coefficient for salts and depends on how many ions dissociate in solution, *R*_g_ is the ideal gas constant, and *m* is the molality of the solution (*16*, *38*). Pressure and temperature desalination processes have an osmotic pressure gradient driving flow in the negative direction (from the permeate to the feed) caused by salinity of the feed solution (*39*). Concentration-driven processes have an osmotic pressure difference driving flow in the positive direction because the driving force from the draw solution overcomes the salinity of the feed solution (*16*, *39*, *40*).

The driving force from a temperature difference is explained by the thermo-osmotic pressure term of Eq. 1: $\frac{Q^{*}}{V_{w}T}\Delta T$ (*23*, *31*, *35*, *41*, *42*). This thermo-osmotic pressure term for liquid- and air-filled membranes can be explained by the flux-force relationship between heat and mass flux. The derivation of temperature-driven mass transport is further explained in the Supplementary Materials, as well as in previous works by Kedem and Kjelstrup (*23*, *29*). An important characteristic of the thermo-osmotic pressure is that, unlike hydraulic and osmotic pressure, the magnitude and sign of the term depends on the type of membrane. This dependence is manifested in the Q^*^ term that represents the heat transferred per mole of fluid permeating across the membrane. In air-filled membranes, Q^*^ is approximately equal to the enthalpy of vaporization that represents the latent heat carried across the membrane by permeating vapor molecules. It is important to note that Q^*^ is only approximately equal to the enthalpy of vaporization because of this value being offset by a kinetic factor owning to a temperature discontinuity derived from kinetic theory (*43*, *44*). This difference in value has also been shown more recently in molecular dynamic simulations (*41*). In liquid-filled membranes, the direction of heat transport has been shown to depend on membrane material. In the case of hydrophilic liquid-filled membranes, there is a decrease in enthalpy when water sorbs into the membrane, which results in heat being released. The opposite effect occurs in the case of hydrophobic liquid-filled membranes, where water has a positive enthalpy of sorption. This increase in enthalpy causes water to release heat on the hot side and absorb heat on the cold side, yielding a positive Q^*^ for hydrophobic membranes and a negative Q^*^ for hydrophilic membranes. Because the thermo-osmotic pressure generated across the membrane depends on Q^*^, water flux typically moves from the cold side to the hot side through hydrophilic membranes and from the hot side to the cold side through hydrophobic membranes (*23*).

### Comparative driving forces from pressure, concentration, and temperature

**Fig. 2.**
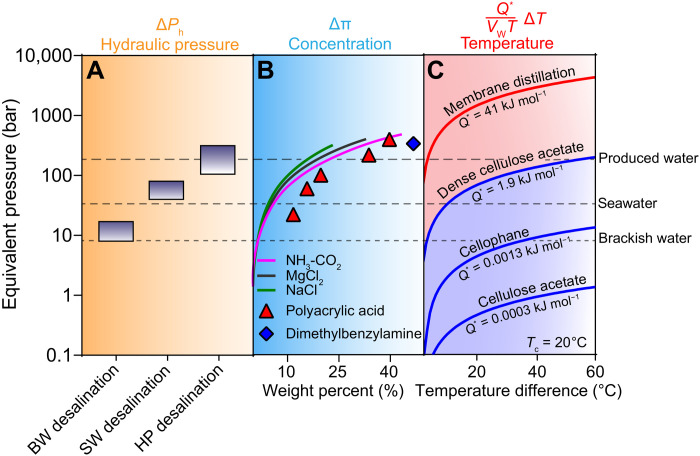
Driving force from gradients in pressure, concentration, and temperature expressed as an equivalent pressure (that is, a hydraulic, osmotic, or thermo-osmotic pressure). (**A**) Hydraulic pressure is shown for ranges of pressures commonly used for brackish water (BW) desalination, seawater (SW) desalination, and high-pressure (HP) desalination. (**B**) Osmotic pressure values are shown as a function of solute weight percent using established correlations for salts and experimental data for higher–molecular weight draw solutes (*16*). (**C**) Thermo-osmotic pressure was calculated using heat of transport, Q^*^, values for distillation and dense polymer membranes with a permeate temperature of 20°C (*23*). Representative equivalent pressures corresponding to the osmotic pressures of brackish water, seawater, and produced water are shown with the dashed horizontal lines.

Using the quantitative framework provided by Eq. 1, the driving forces from pressure, concentration, and temperature are compared using units of equivalent pressure in Fig. 2. In each system, the driving force is shown in the positive direction (feed to the permeate solution). Representative values for the osmotic pressure of brackish water (9 bar), seawater (26 bar), and oil- and gas-produced water (200 bar) are also shown as dashed horizontal lines. The driving force of a process must exceed the osmotic pressure of these feed solutions for desalination to occur.

The driving force resulting from a difference in pressure is typically created by an applied hydraulic pressure generated by a pump and can be varied to suit a given feed solution. Representative hydraulic pressure ranges for brackish water, seawater, and high-pressure desalination systems shown in Fig. 2A are based on experimental RO data in the literature (*45*). Because brackish water contains lower salt concentrations than seawater (2000 to 5000 and 33,000 to 37,000 mg liter^−1^, respectively), a lower pressure is required for brackish water desalination than for seawater desalination (*4*). Typical operating pressures for brackish water and seawater desalination are 5 to 30 bar and 40 to 80 bar, respectively (*46*). Recent studies have demonstrated high-pressure RO systems that can withstand feed pressures up to 300 bar, allowing for RO-based treatment of high-salinity brines (*46*). While these high-pressure RO systems are capable of desalinating high-salinity feed waters, there are many design constraints that must be addressed, including high-pressure module materials and pumps that may severely increase the cost of operation. Because these design constraints have yet to be overcome, high-pressure RO systems have not seen large-scale implementation in practice.

The osmotic pressure depends on the solute concentration, and the maximum osmotic pressure is restricted by a solute’s solubility in water (*16*, *47*). The solutes shown in Fig. 2B represent draw solutions commonly used in FO studies, comprising of inorganic salts (including thermolytic salts), organic molecules, and polymer-based draw solutes that, at high weight percent, are capable of generating osmotic pressures greater than 300 bar (*46*, *48*). Draw solutions are chosen on the basis of their diffusivity and ability to generate high osmotic pressures at low viscosities (*16*, *49*). The limit of osmotic pressure generated by the saturated draw solutions is approximately 500 bar. This high osmotic pressure facilitates the desalination of higher salinity brines than pressure-driven processes. However, it is undesirable for the weight percent of the draw solution to be too high as this typically results in increased pumping costs as the viscosity of the solution increases (*16*, *50*).

The thermo-osmotic pressure gradient generated by a temperature difference depends on the heat of water transport (Q^*^) through a given material (*23*). MD uses a difference in vapor pressure across the membrane brought on by an elevated temperature on the feed side of the membrane (*28*). The high driving force in MD, represented by an equivalent pressure in Fig. 2C, is due to water’s relatively large latent heat of vaporization (41 kJ mol^−1^), which approximately corresponds to the Q^*^ value in Eq. 1: $\frac{Q^{*}}{V_{w}T}\Delta T$ (*51*). The equivalent pressure generated by a feed temperature of 80°C and a permeate temperature of 20°C (i.e., a difference of 60°C) is more than 4000 bar, meaning that MD has a much higher driving force than pressure- or concentration-driven systems, can overcome a much higher osmotic pressure difference, and can thus be used to concentrate high-salinity brines to saturation concentrations (*52*, *53*). The high driving force also explains why the MD flux is often observed to be weakly dependent on salinity. We note that the thermo-osmotic driving force in MD can be translated to a difference in vapor pressure, a more common expression for the driving force for vapor transport in the literature.

Compared to all other driving forces, that which is generated by a temperature difference in liquid-filled membranes is relatively small because the heat of transport (Q^*^) tends to be low (*54*, *55*). For dense polymer membranes, typical Q^*^ values range from 0.0003 to 1.9 kJ mol^−1^ (*23*). The highest of these values (1.9 kJ mol^−1^), measured for dense cellulose acetate membranes, corresponds to an equivalent pressure of approximately 200 bar generated by a temperature difference of 60°C. Because the heat of transport in liquid-filled membranes is more than an order of magnitude lower than the latent heat of vaporization, TO has a correspondingly lower driving force than MD. The low heat of transport in liquid-filled membranes is due to the lack of a gas-liquid phase change that occurs during intrapore transport through the membrane phase. Because the values for heat of transport in liquid-filled systems are very low, accurate measurement has been difficult and a wide range of values are described in the literature (*23*).

## WHAT RESISTANCES OCCUR DURING TRANSPORT?

To evaluate mass transport rates in desalination processes, an accurate description of the resistances that occur during transport is needed in addition to knowledge of the driving forces from pressure, concentration, and temperature differences described in the previous section. In this section, we will highlight the key resistances that occur in liquid- and air-filled membranes. In the case of liquid-filled membranes, we focus specifically on dense polymer materials that are most commonly used for desalination processes (e.g., polyamide thin-film composite membranes). We then describe how these resistances are related to liquid and vapor permeability coefficients and introduce an expression that allows for the comparison of permeabilities of both types of membranes.

### Sorption and diffusion in dense polymer liquid-filled membranes

Operating under the assumptions of a constant transmembrane pressure and chemical equilibrium at the membrane interface, the solution-diffusion model has been used as the foundation for describing molecular transport in liquid-filled dense polymer membranes (*24*). The basis for solution-diffusion is that in order for any species to permeate across the membrane, that species must first partition into the membrane phase and then diffuse through a dense polymer network (*26*). Although the validity of some aspects of the solution-diffusion model has recently been questioned, the general principles still hold and will be used in this analysis to frame liquid-filled transport in dense polymer membranes (*56*, *57*). Resistances in dense polymer membranes can be described by *A*_l_, or the permeability coefficient for liquid-filled membranes, originally derived from the solution-diffusion model

$$A_{l}=\frac{KD}{\delta }$$ (2)

where *K* is the sorption coefficient, *D* is the diffusion coefficient, and δ is the thickness of the membrane active layer. The permeability coefficient is the inverse of the total resistance and thus a measure of the ease of both the sorption and diffusion steps that occur in transmembrane permeation through dense polymer films (*24*, *26*, *57*). The permeability value can vary depending on the species being transported and the properties of the membrane. Solutes with higher permeabilities result in lower water/solute selectivity, defined as the ratio of water permeability to solute permeability.

Sorption of a species into a polymer structure is the first resistance encountered in liquid-filled membrane permeation. The sorption coefficient, *K*, relates the concentration of a species inside of the membrane polymer phase to the concentration in aqueous solution phase: $K=\frac{c_{m}}{c_{b}}$ where *c_m_* and *c_b_* are the concentrations inside the membrane and in the feed solution, respectively. A large sorption coefficient for a species indicates that little resistance is experienced when the species sorbs into the polymer phase (*58*). The sorption coefficient for water in a polymer is proportional to the volume fraction of dissolved water. The volume fraction of water can be increased by increasing the hydrophilicity of the membrane that is typically done by increasing the number of charged functional groups present within the membrane (*57*, *58*).

Diffusion resistances are encountered once water molecules sorb into the membrane phase. A membrane’s water uptake can be used to determine the polymer’s average free volume, *v*_f_, a parameter used to estimate the diffusion coefficient of the membrane: $D=a\exp\left(-\frac{b}{v_{f}}\right)$ where *a* and *b* are adjustable parameters relating to a species’ size and *v*_f_ is taken to be proportional to the water sorption coefficient for dilute feed solutions (*57*, *59*). Although water content plays a significant role in diffusion through polymer membranes, it alone is not sufficient in explaining transmembrane permeability. Ionizable functional groups found on the membrane surface and inside the membrane can both facilitate water sorption and hinder intramembrane transport (*60*, *61*).

**Fig. 3.**
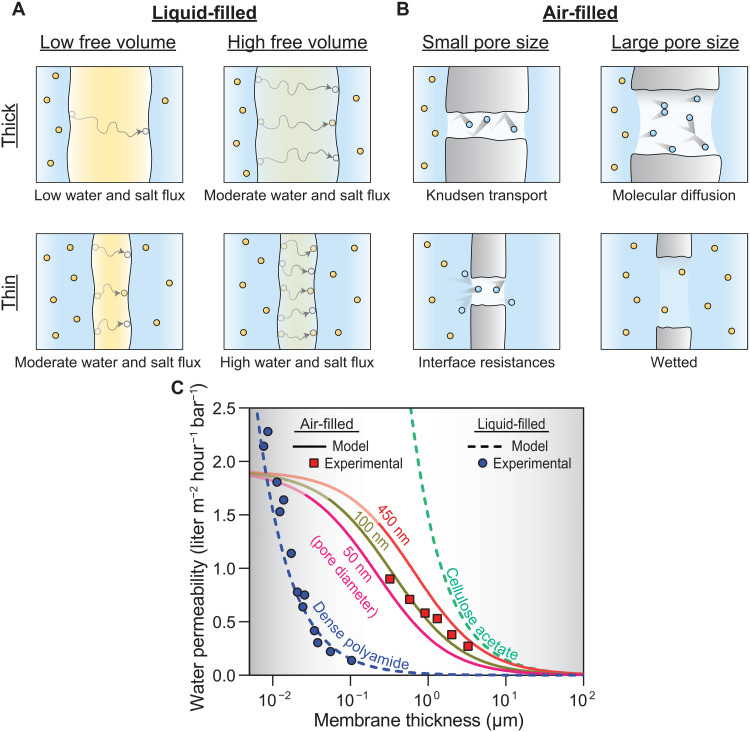
Transmembrane water permeability of liquid- and air-filled membranes. (**A**) Illustrations of high- and low-resistance dense polymer membranes based on active layer structure and thickness. A more dense polymer structure is shown in yellow with a looser polymer structure shown in green. (**B**) Dominant resistances for distillation membranes based on thickness and pore size. Pore wetting in thin membranes with large pore sizes is also shown. (**C**) Water permeability as a function of thickness for liquid- and air-filled membranes. Permeability in air-filled membranes assumes a porosity of 1. Experimental results are included from hydrophobic porous alumina with an average pore diameter of 71.8 ± 23.9 nm and highly cross-linked polyamide formed by molecular layer-by-layer deposition to confirm air- and liquid-filled membrane permeability trends, respectively (*25*, *62*, *63*).

Dense polymer membranes span a wide range of water permeability values depending on their material properties. Figure 3A depicts how membrane permeability and selectivity are affected by the degree of free volume and thickness of membranes (*62*–*64*). For membranes with greater free volume, the diffusion coefficient (and hence permeability) increases, and water-salt selectivity typically decreases. This phenomenon is attributed to the permeability-selectivity trade-off, where increases in water permeability result in disproportionately high increases in salt permeability. Reducing the thickness of the membrane also results in an increase in both water and salt permeability (*63*, *65*).

### Evaporation and diffusion in air-filled membranes

The porous and hydrophobic nature of air-filled membranes results in the formation of an air gap between the feed and permeate liquid streams (*17*). This air gap allows for mass transport through the membrane to occur in the gas phase as water molecules evaporate on the feed liquid-vapor interface, diffuse through the air gap, and then condense on the permeate liquid-vapor interface (*25*, *66*, *67*). Analyzing mass transport in air-filled membranes involves the use of the Dusty-Gas Model and Stefan-Maxwell equation with the following assumptions: The air gap contains a binary mixture of air and water vapor, air is stagnant inside of the pores, flow is one-dimensional, and contributions from viscous flow can be neglected. The Dusty-Gas Model has been applied to transport through porous media and is a well-established model for gas transport (*68*–*70*). In addition, the assumptions made here have been applied in previous work to accurately simplify the expression for vapor permeability in air-filled membranes (*17*, *25*). Mass transport of water through the membrane can be described by gas transport models where the volumetric water flux, *J*_w_, is determined by

$$J_{w}=\frac{\varepsilon }{\rho }\sqrt{\frac{M_{w}}{2\pi RT}}\frac{1}{R_{t}+R_{i,f}+R_{i,p}}\Delta P_{v}=\frac{B_{w}}{\rho }\Delta P_{v}$$ (3)

Where ε is the membrane porosity, ρ is the density of liquid water used to convert mass flux to volumetric flux, *M*_w_ is the molar mass of water, *R* is the universal gas constant, *T* is the average temperature of the air gap, *R*_t_ corresponds to the transmission resistances, and *R*_i,f_ and *R*_i,p_ correspond to the interfacial resistances that occur on the feed and permeate sides of the membrane, respectively, and ∆*P*_v_ is the partial vapor pressure difference across the membrane. (*17*, *28*, *66*, *71*). *B*_w_ is the vapor permeability coefficient, a proportionality factor that relates flux to a partial vapor pressure difference; the inverse of *B*_w_ is the vapor transport resistance that includes contributions from both transmission and interfacial resistances. Transmission resistances, *R*_t_, are the resistances that molecules encounter as they diffuse through the membrane and are influenced by the membranes pore properties, such as radius and length, as well as the characteristics of the gas within the pores (*17*, *25*, *71*). Interfacial resistances, *R*_i_, occur on the vapor-liquid interfaces and stem from the reflection of water molecules at both interfaces that inhibit the required phase changes—evaporation on the feed and condensation on the permeate—that the water molecules undergo to transport through the air gap (*71*).

Transmission resistances are determined by the friction forces that are exerted upon molecules as they travel through the membrane pore and interact with other gas molecules (molecular diffusion) or the walls of the membrane (Knudsen diffusion) (*17*, *25*, *71*). The transmission resistances, *R*_t_, are defined as

$$R_{t}=\frac{\left(1-\frac{P_{v,0}}{P_{t}}\right)u_{w}\delta }{4D_{wa}}+\frac{1}{\eta }$$ (4)

where *P*_v,0_ is the partial vapor pressure of water at the average temperature inside of the air gap, *P*_t_ is the total pressure of gas in the membrane pores, *u*_w_ is the mean molecular speed of water vapor, *D*_wa_ is the diffusion coefficient of water in air, and η is transmission probability (*66*). It is important to note that viscous contributions are considered negligible for vapor transport through a pore when compared to contributions from molecular and Knudsen diffusions (*17*, *71*, *72*). The first term of Eq. 4 that includes *D*_wa_ quantifies molecular-diffusion resistances that occur because of collisions between molecules in the gas phase. Molecular-diffusion resistances can be minimized by decreasing the thickness of the membrane or lowering the air pressure in the pore. The second term in Eq. 4 that includes η describes Knudsen resistances associated with collisions with the pore walls (*71*, *73*). Here, the transmission probability term, η, describes the probability of a gas molecule leaving one liquid-vapor interface of the membrane and arriving at the liquid-vapor interface on the other side of the membrane (*71*, *74*). Previous studies have quantitatively related the transmission probability to length and radius. As the pore length increases and the pore radius decreases, the transmission probability decreases that leads to an increase in transmission resistance.

Interfacial resistances, *R*_i_, in air-filled membranes are associated with phase changes that occur on the two liquid-air interfaces, evaporation of water at the feed-vapor interface and condensation on the distillate-vapor interface. On the distillate-vapor interface, resistance occurs because not all impinging gas molecules will condense, but some will instead reflect back into the vapor-filled pore (*66*, *71*). On the feed-vapor interface, similar resistances occur because not all liquid water molecules impinging on the interface will evaporate into the gas phase. From this, *R*_i_ can be described as

$$R_{i}=\frac{1-\sigma (T)}{\sigma (T)}$$ (5)

where σ(*T*) is the condensation coefficient and is defined as the probability of a vapor molecule impinging on an interface being absorbed into the bulk liquid (*71*, *75*). The condensation/evaporation coefficient is used to define the interfacial resistances experienced by water in an air-filled membrane, but accurate experimental determination have been challenging and prone to error (*75*, *76*). As a result, there are a wide range of evaporation and condensation coefficients found in the literature, with most values falling between 0.1 and 1.0 (*25*, *75*). The uncertainty in these measurements limits our ability to accurately define the overall vapor permeability in thin air-filled membranes.

Referring to Eqs. 3 and 4, high vapor permeabilities can be achieved in air-filled membranes if the air gap is thin. However, while these thin selective layers improve the permeability of air-filled membranes, they also increase the risk of membrane failure via pore wetting. As illustrated in Fig. 3B, wetting can occur when the grand potential of the wetted state is less than the grand potential of the fully dried state, causing capillary condensation (or pore wetting) and compromising the selectivity of the air gap that results in membrane failure (*71*, *77*). Therefore, understanding how physical parameters of the membrane can prevent pore wetting is critical for the application of air-filled membranes in high-salinity and more complex feed waters. A thermodynamic wetting criterion based on the geometry of each meniscus, traction forces due to surface tension, and an external hydraulic pressure can be used to determine the membrane thickness and pore size in which the two liquid-vapor menisci present in air-filled membranes spontaneously wet

$$\frac{l}{r}>\frac{2}{3(\cos\theta -\cos\alpha )}\left[\frac{1}{1+\sin\theta }+\sin\theta \right]$$ (6)

where *l* is the length of the pore, *r* is the pore radius, α is the equilibrium contact angle formed between a drop of liquid water and the solid membrane surface, and θ is the geometric angle between the liquid-vapor interface and a line normal to the interface that depends on the mechanical equilibrium established between the hydraulic pressure drop across the membrane and the traction force from surface tension (*71*). The geometric angle can be determined using the Young-Laplace equation: $\cos\theta =\frac{-r\Delta P}{2{\beta \gamma }_{LV}}$ where β is a geometric coefficient related to pore geometry and surface roughness and γ*_LV_* is defined as the surface energy between the liquid and gas phases (*71*, *78*). This criterion provides information on the kinetic barrier that is needed to prevent pore wetting and reduce the likelihood of wetting to occur. From this criterion, it can be concluded that for low-pressure air-filled membrane applications such as MD and OD, the minimum pore length increases linearly with the pore radius. For high-pressure applications, the minimum pore length must be significantly larger to overcome the effects of an applied pressure on liquid penetration into the membrane pore. The minimum pore length and radius that is derived from this criterion puts limits on the overall water permeability that is achieved with air-filled membranes.

Pore wetting in air-filled membranes can also occur when an applied pressure overcomes the capillary pressure of the hydrophobic pore causing liquid penetration into the air gap. The liquid entry pressure (LEP) can be derived from the Young-Laplace equation

$$\mathrm{LEP}=-\frac{2\beta \gamma_{LV}\cos\alpha }{r_{\max}}$$ (7)

where *r*_max_ is the maximum membrane pore radius. Equation 7 depends on the balance of mechanical force exerted by an applied pressure against the capillary force of the membrane and surface tension of the liquid penetrant (*71*, *78*). Because LEP does not consider the thermodynamic criteria for spontaneous pore wetting, it is possible for wetting to occur even when the hydraulic pressure applied is less than the LEP of the membrane as described in Eq. 6. It is important to note that the LEP is lowered for feed solutions containing surfactants or other surface tension lowering compounds through the γ*_LV_* term in Eq. 7. While this can prove to be problematic in treating more complex feed waters, this type of wetting can be mitigated by Janus and omniphobic membrane designs (*79*–*81*).

### Comparative permeability in liquid- and air-filled membranes

Understanding how water permeability in liquid- and air-filled membranes are related to one another allows for comparisons between mass transport resistances across a wide range of membrane materials. On the basis of our analysis and the assumption of a water activity coefficient of unity, which would apply to dilute solutions (less than 1 M) and realistic hydraulic pressures (less than 100 bar), the vapor permeability coefficient used for air-filled membranes, *B*_w_, which relates a partial vapor pressure difference to water flux, can be directly related to the water permeability coefficient in Eq. 1 using the following equation

$$A_{a}=\frac{B_{w}}{\rho }\frac{P_{v,0}V_{w}}{RT}$$ (8)

where *A*_a_ is the permeability coefficient for air-filled membranes and *P*_v,0_ is equilibrium vapor pressure. The $\frac{P_{v,0}V_{w}}{RT}$ factor in Eq. 8 facilitates the conversion of a difference in hydraulic, osmotic pressure, or thermo-osmotic pressure to a difference in partial vapor pressure. For example, the relation of vapor pressure to hydraulic and osmotic pressure described by Lee and Karnik (and outlined in the Supplementary Materials) is as follows: $\Delta P_{v}=\frac{(\Delta P-\Delta \pi )P_{v,0}V_{w}}{RT}$ where Δ*P*_v_ is the partial vapor pressure difference across the membrane (*66*). Applying this relation to the thermo-osmotic pressure term in air-filled membranes requires the use of the Clausius-Clapeyron relation: $\frac{dP_{v,0}}{dT}=\frac{P_{v,0}\Delta H_{v\mathrm{ap}}}{RT^{2}}$, where ∆*H*_vap_ is the latent heat of vaporization and approximately equal to Q^*^ for air-filled membranes (*82*). Further explanation and application of this relationship to Eq. 1 can be found in the Supplementary Materials.

The water permeability coefficient, *A*, values for liquid- and air-filled membranes can be directly compared using Eq. 8 and are shown as a function of thickness in Fig. 3C. Permeability in liquid-filled membranes is assumed to be inversely proportional to thickness (Eq. 2) and depends on the diffusion coefficient of water within the membrane (*63*, *83*). Water permeability values are shown in Fig. 3C for liquid-filled dense polyamide membranes used in seawater desalination and cellulose acetate membranes made through phase inversion (*62*–*64*, *84*). Dense polyamide, typically used for high salt rejection RO, has a tightly cross-linked polymer network formed by the reaction of trimesoyl chloride and *m*-phenylenediamine monomers. Permeability values are shown on the basis of experimental measurements of water permeability as a function of thickness for dense polyamide membranes fabricated via layer-by-layer deposition (*63*). The high cross-linking densities of dense polyamide make it more difficult for polymers to swell, decreasing water sorption, free volume, and thus permeability in the membrane compared to other polymer materials (*57*). Cellulose acetate membranes are non–cross-linked dense polymer films typically formed by a phase inversion process, resulting in larger voids in the polymer structure that lend it a higher permeability than dense polyamide (*62*). Although dense polymer membranes can, in theory, reach extremely high water permeabilities, the water permeability coefficient of seawater desalination membranes is typically constrained to 0.5 to 2 liters m^−2^ hour^−1^ bar^−1^ because salt rejection decreases to unacceptably low levels at higher water permeabilities due to the aforementioned permeability-selectivity trade-off (*85*).

The permeability for air-filled membranes of different pore sizes is also shown in Fig. 3C as a function of thickness. As thickness decreases, diffusion resistances also decrease resulting in higher permeability (Eq. 4). At low thicknesses (less than 1 μm), the impact of further decreasing the thickness on permeability is lessened because of interfacial resistances (Eq. 5). Furthermore, air-filled membranes become vulnerable to spontaneous pore wetting at low thicknesses (thickness where pore wetting occurs are indicated by semitransparent coloring in the lines for air-filled membranes in Fig. 3C). The thickness at which this wetting occurs scales with the pore radius (Eq. 6). For membranes with larger pore sizes, the criteria for spontaneous pore wetting are reached at higher thicknesses. Experimental values are included to show agreement with the permeability models; however, it should be noted that the results are limited as it is difficult to achieve low thickness active layers for air-filled membranes (*25*).

Intuitively, it could be expected that, at the same thickness, air-filled membranes show lower mass flux rates than liquid-filled membranes because the mass density of water is much lower in the gas phase than the liquid phase. When concentration and partial pressure are converted to weight percent, air-filled membranes contain less (3.0 to 10%) water compared to liquid-filled dense polymer membranes (20 to 30%) (*25*, *57*, *86*, *87*). However, experimental measurements show that air-filled membranes generally have permeability values that fall between those of dense polyamide and cellulose acetate polymers (*25*). This means that, although air-filled membranes have a lower mass density of water in their pores, separation via phase change allows for the use of membranes with large pore sizes (0.01 to 0.5 μm) that reduce the friction experienced by transported water molecules. Liquid-filled membranes require subnanometer free volume elements that likely impart greater frictional resistances due to confinement effects and interactions with functional groups (*57*, *59*, *61*).

Key differences arise between liquid- and air-filled membranes in their limitations at high water permeabilities (Fig. 3C). Dense polymer membranes have permeabilities inversely proportional to active layer thickness and are able to reach far higher permeabilities than air-filled membranes, but increasing water permeability results in disproportionately high increases in the salt permeability, eventually compromising water-salt selectivity (*5*). From our current understanding, air-filled membranes do not show the same trade-off, but their permeability is limited by both interfacial resistances and spontaneous pore wetting at low active layer thicknesses. However, several studies on air-filled membranes have shown results that contradict this permeability limit, indicating that further investigation into interfacial resistance is required (*20*–*22*). For air-filled membranes, a thinner air gap results in an increased probability of membrane failure through pore wetting, and the minimum thickness achievable depends on the pore size of the membrane and the membrane’s hydrophobicity (Eq. 7). This minimum thickness further serves to prevent air-filled membranes from achieving ultrahigh permeabilities.

## HOW DO CONCENTRATION AND TEMPERATURE POLARIZATION AFFECT DESALINATION PERFORMANCE?

From a practical application perspective, water flux is one of the most important performance metrics in any membrane-based desalination process. Water flux is determined as the product of the membrane permeability and driving force, but the water flux estimated on the basis of the ideal driving force (i.e., the bulk chemical potential difference) only serves as a theoretical upper limit that can never be achieved in a practical process. A fair and insightful comparison of desalination performance between processes with different driving forces must take boundary layer effects into consideration. These boundary layer effects alter the temperature and solute concentration at the membrane-solution interfaces as compared to the bulk solution, ultimately decreasing the effective driving force for water transport and negatively influencing the water flux. In this section, we will summarize the boundary layer effects in different processes and quantitatively evaluate the water fluxes achievable in each system.

### Concentration polarization

**Fig. 4.**
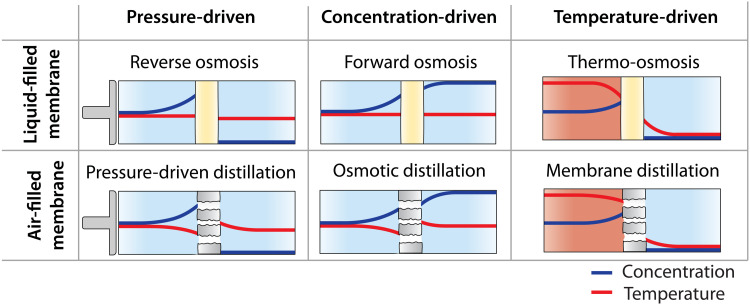
Schematic diagrams of concentration and temperature polarization effects in desalination processes with different driving forces. Blue and red curves are concentration and temperature profiles. Yellow bars and gray bars with gaps represent dense polymer membranes and porous air-filled membranes, respectively. The left side of membrane represents the feed, and the right side represents the permeate (or draw solution for concentration-driven processes).

Concentration polarization (CP) describes a phenomenon in which the solute concentration at the membrane surface differs from that of the bulk solution. In all desalination processes using salt-rejecting membranes, solutes on the feed side cannot pass through the membrane and build up within the boundary layer as water permeates, leading to a higher concentration at the interface than in the bulk solution (Fig. 4). The concentration gradient formed at the membrane surface drives diffusion of solutes from the membrane surface to the bulk feed solution, but this diffusion is counteracted by the advective salt flux toward the membrane caused by water flow. The extent of CP is affected by the permeate flux, the hydrodynamics of the feed stream, and the diffusion coefficient of the solute. A higher permeate flux results in a more severe CP effect. Increasing crossflow velocity of the feed stream can enhance the mixing at the membrane interface and promote mass transfer in the boundary layer, reducing the CP effect. A higher diffusion coefficient of the solute reduces the impact of CP due to increased mass transfer of the solute from the membrane interface to the bulk solution.

In concentration-driven processes (i.e., FO and OD), CP also occurs on the draw solution side as water permeates across the membrane and dilutes the draw solution, resulting in dilutive CP that lowers the concentration at the membrane surface as compared to the bulk draw solution (Fig. 4, middle column). For asymmetric membranes, the porous support layer acts as an unstirred boundary layer that hinders the solute diffusion from the bulk draw solution and leads to more severe CP, known as internal CP (*88*). Similar to external CP, the salt flux across the porous support comprises diffusive and advective components. Although external CP can be attenuated by better hydrodynamic conditions, internal CP is more difficult to mitigate as the mass transfer is primarily dictated by the unstirred layer inside the support. The effective distance that solutes must diffuse through the stagnant support layer is characterized by the structural parameter (*S* = τδ_s_/ε) that groups support layer thickness (δ_s_), porosity (ε), and tortuosity (τ) (*89*, *90*). Severe internal CP leads to a marked reduction of the effective driving force in FO and OD processes and thus reduces the permeate flux.

### Temperature polarization

Similar to CP, temperature polarization (TP) is a phenomenon in which membrane surface temperature is different from that of the bulk solution because of heat transfer across the membrane. The heat flux across the membrane consists of the heat associated with water transport and conductive heat transfer. Although the effect of TP can vary widely between processes, it often reduces the surface temperature on the feed side and increases the surface temperature on the permeate side resulting in a lower net water flux (Fig. 4).

In temperature-driven processes, TP can severely reduce the water flux because the heat flux from both water transport and conductive heat transport work to decrease the temperature difference across the membrane. Mitigation of TP in temperature-driven systems is possible by increasing crossflow velocity on both sides of the membrane to enhance the heat transfer efficiency in the boundary layers. TP can also be reduced by using more thermally insulating membranes to limit the conductive heat flux, which is proportional to the membrane thermal conductivity and inversely proportional to membrane thickness. Air-filled membranes are more insulating than liquid-filled membranes because air (0.025 W m^−1^ K^−1^) has an order of magnitude lower thermal conductivity than water (0.6 W m^−1^ K^−1^) and typical polymeric materials (0.2 W m^−1^ K^−1^) (*91*). Air-filled membranes are also much more porous and typically thicker than liquid-filled membranes, further increasing their thermal insulation. These properties result in air-filled membranes showing notably less severe impacts from TP than liquid-filled membranes in temperature-driven processes.

In pressure- and concentration-driven processes, the effect of TP on the water flux is generally less severe than in temperature-driven processes. In these systems, detrimental heat transfer occurs because of the heat of transport of water through the membrane, but the detrimental heat transfer is counteracted by conductive heat transfer. Heat transfer due to the water flux in air-filled membranes is equivalent to the latent heat of evaporation (41 kJ mol^−1^) and is therefore much higher than that of liquid-filled membranes (3 × 10^−4^ to 2 kJ mol^−1^). Air-filled membranes are also more thermally insulating than liquid-filled membranes, preventing beneficial conductive heat transfer from occurring. Thus, air-filled membranes can show substantial effects from TP in pressure- and concentration-driven systems whereas the impact of TP on liquid-filled membranes is typically negligible.

### Combined impact of boundary layer effects on water productivity

Water flux models (details in the Supplementary Materials) accounting for boundary layer effects can be used to directly compare the flux performance of different processes. Here, we simulate the seawater desalination flux of different processes as a function of water permeability. For pressure-driven processes, 70 bar of applied pressure is simulated, a typical operating pressure that is suitable for overcoming the brine osmotic pressure of seawater desalination with 50% recovery. For concentration-driven processes, a draw solution of 3.0 M NaCl is simulated, which generates 146 bar of osmotic pressure at a viscosity near that of pure water. For temperature-driven processes, the feed stream is 60°C, representing the utilization of low-grade heat for thermal-based desalination, and the permeate is 20°C. Complete salt rejection is assumed for air-filled membranes while an empirical correlation between salt and water permeability is assumed for liquid-filled membranes on the basis of experimental data for cross-linked polyamide membranes in the literature (see the Supplementary Materials for further information) (*92*). To ensure the PD membrane is not wetted under high pressures, membrane pore size is maintained at 10 nm, and the water permeability is tuned by altering the membrane thickness. Water permeability of the OD and MD membranes was tuned by adjusting the membrane pore size and thickness simultaneously while satisfying the nonwetting aspect ratio. Both FO and OD membranes were assumed to have a porous hydrophilic support layer facing the draw solution with a structural parameter of 200 μm.

**Fig. 5.**
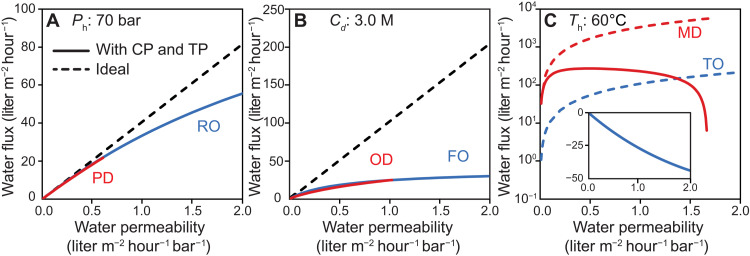
Water flux of all six processes with and without polarization effects. (**A**) Water flux of RO and PD with and without polarization effects. The applied hydraulic pressure is 70 bar. The air-filled membrane has a pore diameter of 10 nm to prevent wetting. (**B**) Water flux of FO and OD with and without polarization effects. The draw solution is a 3 M NaCl solution, and it is assumed that FO and OD membranes have the same support layer with a structural parameter of 200 μm. (**C**) Water flux of TO and MD with and without polarization effects. The feed stream temperature is 60°C, and the permeate stream is 20°C. The inset shows the negative flux of TO with polarization effects. The mass transfer coefficient at the membrane surface is 150 liters m^−2^ hour^−1^, and heat transfer coefficient is 20,000 W m^−2^ K^−1^. It is assumed that the liquid-filled membrane has a porosity of 0.05, and the air-filled membrane has a porosity of 0.8. Tortuosity, τ, is related to porosity, ε, using the following equation: τ = ε^−0.5^. The air-filled membrane’s aspect ratio is simulated so it always meets the criteria for nonwetting (Eq. 6). The feed stream is a 0.6 M NaCl solution.

Water fluxes of pressure-driven processes (i.e., RO and PD) with a constant hydraulic pressure (i.e., 70 bar) were calculated as a function of water permeability accounting for boundary layer effects (Fig. 5A). The results show that water fluxes in RO and PD were similar even when accounting for nonideal polarization effects. Increases solute concentration at the membrane surface of the feed side due to CP were substantial and nearly identical in both processes. TP in RO was negligible because the heat of transport in liquid-filled membranes is minimal, and the membrane is highly thermally conductive. TP in PD was also not substantial because membranes suitable for PD must have an extremely thin air layer (less than 1 μm thick), in which case the heat conduction across the membrane matrix is efficient enough to hinder the buildup of a large interfacial temperature difference. With increasing water permeability, the water flux deviates more from the ideal flux because of increasing impacts of CP. Boundary layer effects reduced the water flux by 13.6% in both RO and PD at a water permeability of 0.6 liters m^−2^ hour^−1^ bar^−1^. RO membranes can reach higher permeabilities than PD because they are not constrained by wetting conditions (see Fig. 4) and, therefore, showed both higher fluxes and higher flux reductions due to CP at increasing permeabilities, with a flux reduction of 31.9% observed at a water permeability of 2.0 liters m^−2^ hour^−1^ bar^−1^.

Water fluxes of concentration-driven processes (i.e., FO and OD) with a constant draw solution concentration (i.e., 3.0 M, equivalent to 146-bar osmotic pressure) were simulated with boundary layer effects (Fig. 5B). Simulation results show that CP effects are severe and exacerbated by internal CP in the membrane support. Similar to pressure-driven processes, CP becomes more severe when increasing water permeability. Boundary layer effects reduce water flux by 78.2% in OD and 79.2% FO at a water permeability of 1.0 liter m^−2^ hour^−1^ bar^−1^. FO, which can reach higher permeabilities, yields a flux reduction of 87.7% at a water permeability of 2.0 liters m^−2^ hour^−1^ bar^−1^. TP in the OD process increases with membrane thickness and thus accounts for the smaller flux as compared to FO at the low water permeability range. For example, OD water flux is 12.6% lower than FO at a water permeability of 0.5 liters m^−2^ hour^−1^ bar^−1^.

Water fluxes of temperature-driven processes (i.e., MD and TO) with a constant bulk temperature difference (i.e., 60°C hot temperature and 20°C cold temperature) were calculated (Fig. 5C). The ideal water flux (i.e., without polarization effects) of MD is over an order of magnitude higher than TO, due to the much higher heat of transport (i.e., 41 kJ mol^−1^) across the membrane in MD that yields a higher driving force. In terms of driving force reduction, CP in MD has a negligible contribution compared to the large driving force created by the temperature difference. In contrast, TP notably reduces water flux in MD and results in a nonmonotonic flux dependence on water permeability. For a commercial MD membrane with a pore size of 0.45 μm and a thickness of 150 μm, the water flux with boundary layer effects is 20% lower than the ideal flux, while for a thinner MD membrane with ~10-μm thickness, the water flux reduction due to boundary layer effects can be as high as 87%. A thinner MD membrane suffers from more severe TP effects although its vapor permeability is higher, resulting in an overall effect of reduced water flux at high permeabilities. Compared to MD, TO suffers from far more severe polarization effects, which result in the actual driving force from the temperature difference being too small to overcome the osmotic pressure of the feed solution (Fig. 5C, inset). Thus, the water flux is negative in TO (i.e., water transports from the dilute stream to the feed stream), and TO cannot be practically used for seawater desalination applications.

## WHICH APPLICATIONS ARE DIFFERENT MEMBRANE TYPES AND DRIVING FORCES WELL-SUITED FOR?

In this section, desalination processes with liquid-filled and air-filled membranes under different driving forces are compared in terms of their practical effectiveness in desalination. The discussion uses the preceding quantitative analysis to gain insights into advantages and disadvantages of each distinct process. Energy efficiency, capability of treating high-salinity feed solutions, and water-salt selectivity are considered when evaluating the strengths and weaknesses of each system.

### Pressure-driven processes

**Fig. 6.**
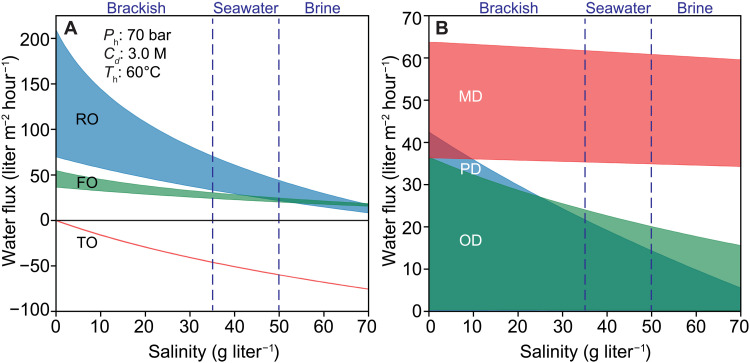
Brine tolerance of desalination processes. Water flux of (**A**) liquid-filled membranes and (**B**) air-filled membranes as a function of feed salinity. Water flux ranges are shown for RO and FO membranes with permeabilities from 1 to 3 liters m^−2^ hour^−1^ bar^−1^. The water permeability of TO membranes is 2 liters m^−2^ hour^−1^ bar^−1^. Heat of transport of TO membranes varies from 0.3 to 2000 J mol^−1^. Upper limits of water flux are shown for optimized PD membranes with a pore size of 10 nm and thickness of 50 nm (water permeability coefficient approximately equals 0.6 liters m^−2^ hour^−1^ bar^−1^), OD membranes with a pore size of 6.8 nm and thickness of 10 nm (1 liter m^−2^ hour^−1^ bar^−1^), MD membranes with a thickness of 90 μm (0.03 liters m^−2^ hour^−1^ bar^−1^). Lower limits for flux in air-filled membranes are shown for membranes with a pore diameter of 0.45 μm and a thickness of 150 μm (0.013 liters m^−2^ hour^−1^ bar^−1^). We assume that FO and OD membranes have the same support layer with a structural parameter of 200 μm. The applied hydraulic pressure for RO and PD is 70 bar. The draw solution for FO and OD is 3 M NaCl (i.e., 146-bar osmotic pressure). The hot stream bulk temperature for MD and TO is 60°C, and the permeate stream is 20°C. We assume that the mass transfer coefficient at the membrane surface is 150 liters m^−2^ hour^−1^, the heat transfer coefficient is 20,000 W m^−2^ K^−1^, liquid-filled membranes have a porosity of 0.05, and air-filled membranes have a porosity of 0.8. Tortuosity is estimated by ε^−0.5^ (*91*, *108*).

Pressure-driven processes (RO and PD) benefit from high efficiency as compared to other separation technologies but are limited to applications that do not require the treatment of brines with prohibitively high salinity (*93*). The high energy efficiency in pressure-driven systems is evident from the previous analysis of boundary layer effects: Unlike concentration- or temperature-driven processes, the driving force from a pressure difference can be used to produce water without being lost to polarization effects. The advantages in energy efficiency are apparent from investigations of RO systems where specific energy consumption has decreased to approximately 2 kWh per cubic meter of product water in the past decades, a value that approaches the thermodynamic limit (e.g., 1.06 kWh m^−3^ at 50% water recovery) (*2*, *93*, *94*). PD systems are expected to share similarly high energy efficiencies as RO. A key limitation that pressure-driven technologies share is the challenges in treating high-salinity brines because typical systems can only reach hydraulic pressures around 80 bar. Figure 6 shows the dependence of water flux on feed salinity simulated for coupon-scale systems. From these simulations, it is evident that the water flux in pressure-driven systems strongly depends on feed salinity. The disadvantage of a limited working pressure range may be overcome through the development of higher pressure systems (*46*, *48*). Development of high-pressure RO can potentially push the operating pressure up to approximately 150 bar, with which a two- to threefold reduction in energy consumption for the desalination of high-salinity brines can be expected compared to thermal technologies (*46*).

A key advantage of RO as compared to PD is the higher achievable water permeability, which can allow for higher water fluxes with the same pressure difference. Air-filled membranes used in PD have permeability limits that are mainly due to transport resistances at the liquid-vapor interface (Eq. 5) and limitations in how thin membranes can be made because of wetting constraints. We note that transport resistances at the liquid-vapor interface have only recently been defined, and a few recent studies have shown water permeabilities in air-filled membranes that exceed those theoretically possible based on current transport models (*20*, *21*, *25*, *95*, *96*). Thus, it may be possible to create high-permeability air-filled membranes that can offer comparable water permeabilities to RO membranes without sacrificing selectivity.

Selectivity and membrane robustness are also key distinguishing features between RO and PD systems. Dense polymer membranes typically used in RO are constrained by the permeability-selectivity trade-off where improvements in membrane water permeability result in a sacrifice of water-solute selectivity (*97*). Polyamide membranes also poorly reject small neutral solutes, like boron, urea, and certain micropollutants. In addition, most dense polymer membranes suffer from performance decay when exposed to oxidizing species (e.g., chlorine and ozone), limiting their lifetime and increasing operation costs (*98*, *99*). Conversely, air-filled membranes are not constrained by the permeability-selectivity trade-off and all nonvolatile solutes, including low–molecular weight contaminants such as boron and urea, can be perfectly rejected. Moreover, air-filled membranes have better oxidation resistance than dense polymer membranes due to the use of robust hydrophobic materials in these systems (*100*, *101*). However, a key challenge of PD membranes and air-filled membranes more generally is the possibility of membrane wetting that can compromise selectivity in long-term operation. These wetting phenomena require further experimental investigation because PD is a nascent technology with few experimental studies (*36*).

### Concentration-driven processes

Processes driven by a concentration gradient (FO and OD) have advantages compared to pressure-driven systems because they can treat higher-salinity waters and have been shown to have lower fouling propensity (*16*). Concentration-driven systems are capable of treating higher salinities than pressure-driven systems because the osmotic pressure of draw solutions can easily exceed 100 bar, notably higher than the pressure that can be applied in conventional and even high-pressure RO systems. The effect of this higher driving force is evident in Fig. 6, where the water flux achievable with FO and OD systems is less affected by the feed salinity than that of pressure-driven systems. Lower fouling propensity has been shown in FO processes because the cake layer formed by foulants is less compact than that formed in a pressure-driven system, and the same advantage can be expected for OD systems as compared to PD (*102*).

A key disadvantage of concentration-driven processes is the lower energy efficiency and more complicated operation than pressure-driven systems (*16*). Standalone concentration-driven processes cannot carry out desalination as the product water needs to be further separated from the diluted draw solution. This separation is inevitably more energy intensive than a standalone pressure-driven system (*16*). The use of thermolytic draw solutes, like ammonia-carbon dioxide, that allow regeneration of the draw solution with low-grade thermal energy provides a possible avenue to reduce electricity consumption (*103*). However, efforts to implement these systems have been stymied by loss of the draw solute via reverse salt flux and the need for energy-intensive draw solution recovery technologies. Concentration-driven processes also suffer from increased losses in their driving force due to polarization effects as compared to pressure-driven systems.

Although FO and OD share many similarities, the different types of membranes used in both processes result in unique advantages and disadvantages. Liquid-filled membranes used in FO generally offer higher permeabilities, which can be advantageous for obtaining higher water fluxes. However, these membranes also suffer from the selectivity limitations mentioned earlier, which are particularly detrimental in concentration-driven systems because reverse salt flux of the draw solution reduces the water flux by exaggerating CP and creates a need for continuous replenishment of draw solutes (*16*). OD membranes offer generally higher selectivity that helps prevent reverse salt flux, but they also poorly reject volatile molecules that may be used in thermolytic draw solutions.

### Temperature-driven processes

The temperature-driven MD process benefits from the highest driving force of any of the processes studied in this analysis but is also limited by a low energy efficiency. The high driving force from MD (up to 3000 bar of equivalent pressure with a pair of working temperatures of 60° and 20°C) allows for treatment of brines up to saturation concentrations and results in the water flux being only weakly dependent on the salinity of the feed (Fig. 6B). However, the high driving force also results in energy consumption one to two orders of magnitude higher than that of pressure-driven systems (*7*). This high energy consumption of thermal technologies is a result of the high latent heat of evaporation of water, which places an extremely high thermal energy cost on the phase change of water required to produce permeate (over 12 times higher than the Gibbs free energy of separation for seawater) (*72*). While heat recovery can reduce energy consumption, there are still inevitable thermal losses in MD. Furthermore, as shown in our preceding analysis on boundary layer effects, MD can suffer severe TP that diminishes most of the driving force resulting from a temperature difference, further limiting the energy efficiency of the process.

Because of the low driving force caused by a small heat of transport and severe TP in liquid-filled polymeric membranes for TO, the thermo-osmotic pressure across membrane interfaces is not large enough for seawater or even brackish water desalination. TO with polymer materials is, therefore, not a promising avenue for desalination. However, molecular simulations have shown novel two-dimensional desalination membranes with a higher heat of transport, like aligned carbon nanotube membranes, may have the potential for brackish water and seawater desalination given a reasonable temperature difference (*104*, *105*). Further fundamental understanding of TO is required on how heat of transport is affected by membrane material and structure. Experimental evidence of TO with aligned carbon nanotube membranes is necessary to support the claimed capability of desalination with the TO process.

## FUTURE RESEARCH NEEDS AND OUTLOOK

**Fig. 7.**
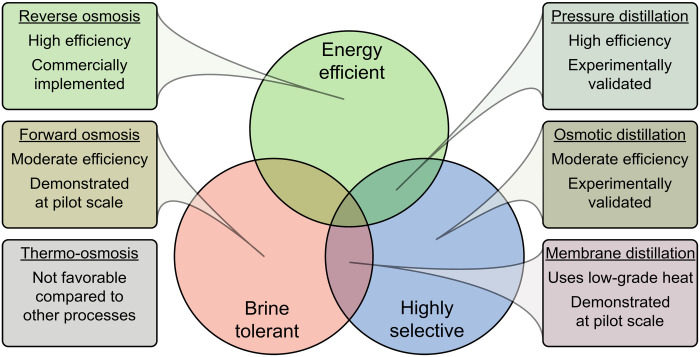
Summary of the advantages and maturity of pressure-, concentration-, and temperature-driven processes with liquid-filled and air-filled membranes.

Desalination processes driven by differences in hydraulic pressure, concentration, and temperature have distinct characteristics that enhance or limit their ability to be used in desalination systems. Figure 7 shows the qualitative advantages and disadvantages of each process, highlighting three main factors when considering a desalination process: energy efficiency, contaminant selectivity, and tolerance to higher-salinity feed solutions. The technical maturity of each process is also indicated.

Pressure-driven desalination methods including PD and RO are inherently energy efficient as compared to concentration- and temperature-driven processes (*7*, *16*, *94*). However, the water output of pressure-driven processes is dependent on the salinity of the feed solution, making these processes less effective at treating high-salinity feed waters. Among the pressure-driven processes, RO is much more technically mature, but PD may have potential owing to the high removal of all nonvolatile compounds possible with air-filled membranes. Concentration-driven processes such as OD and FO are able to treat high-salinity brines but are more energy intensive than pressure-driven systems as draw solution regeneration inherently requires more energy than a direct pressure-driven separation. Temperature-driven MD systems are able to treat high-salinity brines with high selectivity but suffer from lower energy-efficiency than pressure- and concentration-driven systems. TO is not considered advantageous in any scenario currently because of its low driving force and severe flux decline brought on by boundary layer effects.

Several research gaps limit our ability to conclusively analyze mass transport in different desalination processes. For air-filled membranes, mass transport can be accurately modeled for relatively thick (greater than 10 μm) air gaps where Knudsen and molecular diffusions are dominant in determining the overall resistances experienced by water vapor. However, as thickness is decreased, interfacial resistances from phase transitions start to dominate transport resistances; these interfacial resistances have only been described in a few recent studies, and experimental works have shown ultrahigh water fluxes that may go beyond the permeability limits established by existing transport models (*20*–*22*, *75*, *76*). A better understanding of transport in ultrathin air-filled membranes is especially important for PD and OD, which require thin air-filled membranes to reach comparable water permeabilities to state-of-the-art thin-film composite membranes. Further research into vapor transport in thin air-filled membranes is, therefore, needed to better understand performance, especially in the poorly studied PD process.

Much of the preceding analysis on liquid-filled membranes has specifically focused on dense polymer materials due to the wealth of experimental and theoretical data on these membranes. While we expect other materials that separate via steric and electrostatic interactions to behave similarly, there is potential for membrane materials that radically alter mass transport. Such systems using materials like graphene, aquaporins, or artificial channels may follow different transport physics than those of dense polymer membranes, altering the transport resistances for water and moving beyond permeability-selectivity limitations that constrain current dense polymeric membrane materials (*106*). A better understanding of fundamental transport in all liquid-filled systems, including friction and confinements effects, may allow for better predictive modeling of transport that does not rely on empirically determined diffusion and sorption coefficients (*57*, *107*).

The framework presented in this work enables the direct comparison of driving forces, mass transport resistances, and ultimately water flux in desalination processes using salt-rejecting membranes. Future application of the framework presented here to other liquid-filled membranes and air-filled membranes with lower active layer thicknesses will shed light on transport effects that are currently not well understood, allowing for the development of more effective desalination membranes. Incorporation of electrochemical driving forces may further broaden our ability to compare widely different separations processes. Moreover, application to nonaqueous separations or power generation processes will allow us to further elucidate fundamental advantages and disadvantages of various engineered processes.

## Supplementary Materials

### This PDF file includes:

Supplementary Text Table S1 References
