# Supplementary material for: Toward a universal framework for evaluating transport resistances and driving forces in membrane-based desalination processes
Source: Sci Adv. 2023 Jan 4;9(1):eade0413. doi: 10.1126/sciadv.ade0413 (PMC9812388; doi:10.1126/sciadv.ade0413)
Supplement: Supplementary file 1 — Supplementary Text Table S1 References [file sciadv.ade0413_sm.pdf]

Science Advances
AAAS

Supplementary Materials for

Toward a universal framework for evaluating transport resistances and driving forces in membrane-based desalination processes

Kian P. Lopez *et al.*

Corresponding author: Anthony P. Straub, anthony.straub@colorado.edu

Sci. Adv. **9**, eade0413 (2023)
DOI: 10.1126/sciadv.ade0413

This PDF file includes:

Supplementary Text
Table S1
References

Supplementary Text

Relating the transport of heat and mass in membrane-based systems using entropy production

Transport of mass and heat in membrane systems can be related using non-equilibrium thermodynamics allowing for entropy production in the membrane to be expressed as a function of gradients in both chemical potential and temperature:

$$\sigma = J_q \Delta\left(\frac{1}{T}\right) + J_w\left(-\frac{1}{T}\Delta\mu_w\right) \tag{S1}$$

where σ is entropy production, J_q is heat flux, T is temperature, J_w is water flux, and μ_w is chemical potential (*23*, *35*). Gradients in chemical potential relate pressure and concentration to mass flux while a difference in temperature relates to heat flux. An equation relating the transport of both mass and heat can be derived through force-flux relationships, relating differences in chemical potential and temperature to mass and heat flux:

$$J_q = -l_{qq}\left(\frac{\Delta T}{T^2}\right) - l_{qV}\left(\frac{1}{T}\Delta\mu_w\right) \tag{S2}$$

$$J_w = -l_{Vq}\left(\frac{\Delta T}{T^2}\right) - l_{VV}\left(\frac{1}{T}\Delta\mu_w\right) \tag{S3}$$

where l_{ij} are the conductive coefficients with l_{qq} representing Fourier conductivity, l_{vv} correlating to the water permeability, and l_{vq}/l_{qv} relating to the Dufour coefficient (*41*). The Onsager relation $l_{vq} = l_{qv}$ applies allowing for the derivation of water flux in terms of gradients in chemical potential and temperature:

$$J_w = -\frac{Q^* A}{V_w T}\Delta T - \frac{A}{v_w}\Delta\mu_w \tag{S4}$$

where Q^* is the heat of transport (typically equal to a difference in partial molar enthalpy), V_w is the molar volume of liquid water, and A is the water permeability coefficient defined in Eq. 1 of the main text. Under the assumption of a local isothermal condition, chemical potential can be expressed in terms of pressure and concentration:

$$\Delta\mu_w = V_w(\Delta P_h - \Delta\pi) \tag{S5}$$

where ΔP_h and $\Delta\pi$ are the differences in hydraulic and osmotic pressure across the membrane (*29*). Substitution into Eq. S4 allows for water flux to be expressed in terms of pressure, concentration, and temperature:

$$J_w = A\left((\Delta P_h - \Delta\pi) + \frac{Q^*}{V_w T}\Delta T\right) \tag{S6}$$

<u>Directly comparing liquid and vapor permeability coefficients</u>

The correlation between a difference in hydraulic and osmotic pressure across a nanopore, ΔP_h and $\Delta\pi$, and a difference in vapor pressure, ΔP_v , was originally derived by Lee and Karnik:

$$\Delta P_v(P_h, C, T) = \frac{(\Delta P_h - \Delta\pi)V_w}{RT} P_{v,0}(T) \tag{S7}$$

where $P_{v,0}(T)$ is the equilibrium vapor pressure of water at temperature, *T* (*66*). Equating water flux in both liquid- and air-filled membranes allows for the relation between the water permeability coefficient (*A*) used in Eq. 1 and the vapor permeability coefficient (B_w) used to model transport in air-filled membranes; this relation between *A* and B_w is shown in Eq. 8 of the main text. It is important to note that this derivation involves the use of the Kohler equation, Raoult's law for dilute solutions (<1M), and the assumption of a hydraulic pressure less than 100 bar. The relationship in Eq. S7 along with Eq. 8 from the main text can be applied to the universal water flux equation for each driving force where pressure-driven and concentration-driven processes are shown in Eq. S8.

$$J_w = A[\Delta P_h - \Delta\pi] = \frac{B_w}{\rho}\frac{P_{v,0}(T)V_w}{RT}[\Delta P_\mathrm{h} - \Delta\pi] = \frac{B_w}{\rho}\Delta P_v(P_h, C) \tag{S8}$$

Eq. S8 shows how with the assumption of pressure and concentration gradients as the only driving forces, Eq. 1 can be applied to both liquid-filled reverse osmosis and forward osmosis as well as air-filled pressure-driven and osmotic distillation processes.

Similarly, assuming a temperature difference to be the only driving force, Eq. S9 shows how applying Eq. 8, the Clausius-Clapeyron relationship, and the Lagrange mean value theorem allows for the conversion of Eq. 1 from describing the water flux in thermo-osmosis to the water flux in membrane distillation.

$$\begin{aligned} J_w &= A\left[\frac{Q^*}{V_w T}\Delta T\right] = \frac{B_w}{\rho}\frac{P_{v,o}(T)Q^*}{RT^2}\Delta T \approx \frac{B_w}{\rho}\frac{P_{v,o}(\mathrm{T})\Delta H_{vap}}{RT^2}\Delta T \\ &= \frac{B_w}{\rho}\frac{dP_v}{dT}\Delta T \approx B_w \Delta P_v(T) \end{aligned} \tag{S9}$$

Concentration polarization and temperature polarization

The magnitude of concentration polarization is quantified using the concentration polarization coefficient (*CPC*) which is defined as the ratio of the interfacial concentration difference (ΔC_m) and the bulk concentration difference (ΔC_b), which is affected by both permeate flux and hydrodynamics of the feed stream:

$$CPC = \frac{\Delta C_m}{\Delta C_b} \qquad \text{(S10)}$$

Film theory is usually applied to estimate the interfacial concentration. For RO, *CPC* is estimated as:

$$CPC_{RO} = \frac{C_{F,m} - C_P}{C_{F,b} - C_P} = exp\left(\frac{J_w}{k_f}\right) \qquad \text{(S11)}$$

where $C_{F,m}$, $C_{F,b}$, and C_P are feed interfacial concentration, feed bulk concentration, and permeate concentration, respectively; J_w is permeate flux; and k_f is the mass transfer coefficient near membrane surface and can be estimated with the Sherwood number (*109*). For PD, *CPC* is estimated as:

$$CPC_{PD} = \frac{C_{F,m}}{C_{F,b}} = exp\left(\frac{J_w}{k_f}\right) \qquad \text{(S12)}$$

For FO, *CPC* is estimated as:

$$CPC_{FO} = \frac{C_{D,m} - C_{F,m}}{C_{D,b} - C_{F,b}} = \frac{C_{D,b}exp\left(\frac{-J_w S}{D}\right) - C_{F,b}exp\left(\frac{J_w}{k_f}\right)}{1 + \frac{B_s}{J_w}\left(exp\left(\frac{J_w}{k_f}\right) - exp\left(\frac{-J_w S}{D}\right)\right)} \frac{1}{C_{D,b} - C_{F,b}} \qquad \text{(S13)}$$

where $C_{D,m}$ and $C_{D,b}$ are draw interfacial concentration and draw bulk concentration, respectively; S is the structural parameter of the support layer; D is the solute diffusion coefficient; and B_s is the solute permeability (*92*, *109*). For OD, *CPC* is estimated as:

$$CPC_{OD} = \frac{C_{D,m} - C_{F,m}}{C_{D,b} - C_{F,b}} = \frac{C_{D,b}exp\left(\frac{-J_w S}{D}\right) - C_{F,b}exp\left(\frac{J_w}{k_f}\right)}{C_{D,b} - C_{F,b}} \qquad \text{(S14)}$$

We note that external CP on draw side is neglected for FO and OD because it is insignificant as compared to the internal CP within the support layer (*109*). For MD, *CPC* is estimated in a manner analogous to TP:

$$CPC_{MD} = \frac{C_{F,m}}{C_{F,b}} = exp\left(\frac{J_w}{k_f}\right) \qquad \text{(S15)}$$

The magnitude of temperature polarization is quantified using the temperature polarization coefficient (*TPC*), which is defined as the ratio of interfacial temperature difference (ΔT_m) and bulk temperature difference (ΔT_b) in temperature driving processes (e.g., MD and TO), which is mainly determined by water flux, membrane thermal conductivity, and bulk stream heat transfer coefficients (*110*, *111*):

$$J_w = \frac{B_w}{\rho}\left(P_v\left(P_{h,f}, C_{f,m}, T_{f,m}\right) - P_v\left(P_{h,p}, C_{p,m}, T_{p,m}\right)\right) \quad \text{(S23)}$$

where B_w is water vapor permeability coefficient and ρ is the density of liquid water (*66*, *71*). $P_v(T, C, P_h)$ is water vapor pressure as a function of temperature, concentration, and hydraulic pressure, and can be further expressed as:

$$P_v(P_h, C, T) = P_{v,0}(T)a_w(C)exp\left(\frac{P_h V_w}{RT}\right) \quad \text{(S24)}$$

The equilibrium vapor pressure, $P_{v,0}(T)$, can be estimated using Antoine's equation:

$$P_{v,0} = exp\left(A - \frac{B}{T + C}\right) \quad \text{(S25)}$$

Where A, B, and C are Antoine coefficients specific to a given substance (*114*). The water activity $a_w(C)$ can be estimated by an empirical equation (*115*):

$$a_w(C) = 1 - 0.03112C - 0.001482C^2 \quad \text{(S26)}$$

For liquid-filled membranes, volumetric water (J_w) and salt (J_s) fluxes can be calculated by:

$$J_w = A(\Delta P_h - \Delta\pi + \frac{Q^*}{V_w T}\Delta T\ ) \quad \text{(S27)}$$

$$J_s = B_s \Delta C_m \quad \text{(S28)}$$

where A and B_s are water and salt permeabilities, respectively, and are correlated by $B_s = 0.0133A^3$; ΔP_h is the applied hydraulic pressure, $\frac{Q^*}{V_w T}\Delta T$ is thermo-osmotic pressure, and $\Delta\pi$ is the osmotic pressure difference (*92*).

<u>Discussion of unexpectedly high mass transport rates in air-filled membranes</u>

The current framework for vapor transport in air-filled membranes claims that thinner membranes result in a reduction in transmission resistances. However, when the air gap (or active layer) thickness is decreased, air-filled membranes suffer from an increase in temperature polarization in membrane distillation as well as more prominent interfacial resistances associated with evaporation and condensation. Thus, the maximum mass transport rate of air-filled membranes can be achieved by optimizing thickness with a theoretical upper limit set by both temperature polarization and interfacial resistances. Although most of the literature for air-filled membranes reports permeabilities within the range of the current framework, it is important to point out that a few studies have shown permeabilities beyond the theoretical upper limit (*20–22*, *80*, *95*, *116, 117*). Such high fluxes may be attributable to cluster evaporation, changes in the enthalpy of vaporization, or unexpectedly high values for the condensation coefficient. Further study of these anomalously high transport rates may demonstrate that the water flux limits defined by conventional theory must be revised.

$$TPC = \frac{\Delta T_m}{\Delta T_b} \quad \text{(S16)}$$

For pressure and concentration driven processes, ΔT_m alone the reflects the degree of TP, as ΔT_b is zero. Using MD as an example, interfacial temperatures can be estimated by solving the following heat transfer equations:

$$J_q = J_v Q^* + \frac{k_m}{\delta_m}\left(T_{H,m} - T_{C,m}\right) \quad \text{(S17)}$$

$$T_{H,m} = T_{H,b} - \frac{J_q}{h_H} \quad \text{(S18)}$$

$$T_{C,m} = T_{C,b} + \frac{J_q}{h_C} \quad \text{(S19)}$$

where J_q and J_v are heat flux and water vapor flux, respectively; Q^* is the heat of transport across the membrane; h_H and h_C are heat transfer coefficients of hot feed stream and cold permeate stream, respectively, and can be estimated with Nusselt number; and δ_m is membrane thickness (*28*). The membrane thermal conductivity, k_m, can be estimated by the following equation:

$$k_m = (1 - \varepsilon)k_p + \varepsilon k_g \quad \text{(S20)}$$

where ε is membrane porosity, and k_p and k_a are thermal conductivity of the polymer matrix and air, respectively (*28*). The general approach also applies to PD, OD, and TO processes. We note that TP is usually not considered in RO and FO processes since the effect is typically negligible. For asymmetric composite membranes with a hydrophilic support layer, the heat transfer coefficient of the stream adjacent to the support layer needs to be modified to capture the internal TP effect:

$$h' = \left(\frac{1}{h} + \frac{\delta_s}{k_s}\right)^{-1} \quad \text{(S21)}$$

where $k_s$and δ_s are thermal conductivity and thickness of the support layer, respectively (*112*). It is important to note that CP and TP can be related through the heat and mass analogy by the following equation:

$$\frac{C_m}{C_b} = \exp\left(\frac{\eta_{th} C_p \Delta T_{TP}}{h_{fg}}\right) * Le^{n-1} \quad \text{(S22)}$$

where η_{th} is the thermal efficiency, C_p is the specific heat, ΔT_{TP} is the temperature across the temperature polarization region, h_{fg} is the enthalpy vaporization, n is the exponent of the Prandtl number in the Nusselt number correlation, and Le is the Lewis number that defined as the ratio of thermal diffusivity and mass diffusivity (*113*).

Flux calculation for air-filled and liquid-filled membranes

For air-filled membranes, volumetric water vapor flux (J_w) can be calculated by:

Table S1. Definition of parameters used in simulating transport through membrane-based desalination processes.

Parameter	Definition	Equation/Method
τ	Ratio of total distance a molecule must travel through a polymer to the polymer film thickness	$\tau = \varepsilon^{-0.5}$ (*118*)
v_f	Volume within the polymer that is not occupied by the polymer matrix	$v_f = K_W v_{F,W} + (1 - K_W) v_{F,p}$ (*5*)<br><br>K_w – Water sorption coefficient<br><br>$v_{f,w}$ – Free volume of pure water<br><br>$v_{f,p}$ – Free volume of dry polymer
σ	Probability of a gaseous molecule impingent on a liquid surface entering the bulk liquid phase	Determined experimentally or through simulations (*25*)
η	Probability of a gas molecule on one side of the pore reaching the other side	$\eta = 1 + \left(\frac{L^2}{4}\right) - \left(\frac{L}{4}\right)\left(L^2+4\right)^{\frac{1}{2}} - \frac{\left[\left(8-L^2\right)\left(L^2+4\right)^{\frac{1}{2}} + L^3 - 16\right]^2}{72L(L^2+4)^{\frac{1}{2}} - 288\ln[L+(L^2+4)^{\frac{1}{2}}] + 288\ln 2}$<br>(*71*)<br><br>$L$ – pore aspect ratio (pore length/pore radius)
a	Adjustable constant	$a = D_s^{\circ} \exp(b)$ (*5*)<br><br>D_s° - self diffusion coefficient in water
b	Adjustable constant related to the size of the species	Determined experimentally (*5*, *57*)

REFERENCES AND NOTES

1. A. Park Williams, E. R. Cook, J. E. Smerdon, B. I. Cook, J. T. Abatzoglou, K. Bolles, S. H. Baek, A. M. Badger, B. Livneh, Large contribution from anthropogenic warming to an emerging North American megadrought. *Science* **368**, 314–318 (2020).

2. M. Elimelech, W. A. Phillip, The future of seawater desalination: Energy, technology, and the environment. *Science* **333**, 712–717 (2011).

3. N. L. Le, S. P. Nunes, Materials and membrane technologies for water and energy sustainability. *Sustain. Mater. Technol.* **7**, 1–28 (2016).

4. L. F. Greenlee, D. F. Lawler, B. D. Freeman, B. Marrot, P. Moulin, Reverse osmosis desalination: Water sources, technology, and today's challenges. *Water Res.* **43**, 2317–2348 (2009).

5. H. Zhang, G. M. Geise, Modeling the water permeability and water/salt selectivity tradeoff in polymer membranes. *J. Membr. Sci.* **520**, 790–800 (2016).

6. J. R. Werber, C. O. Osuji, M. Elimelech, Materials for next-generation desalination and water purification membranes. *Nat. Rev. Mater.* **1**, 16018 (2016).

7. A. Deshmukh, C. Boo, V. Karanikola, S. Lin, A. P. Straub, T. Tong, D. M. Warsinger, M. Elimelech, Membrane distillation at the water-energy nexus: Limits, opportunities, and challenges. *Energ. Environ. Sci.* **11**, 1177–1196 (2018).

8. J. M. Winglee, N. Bossa, D. Rosen, J. T. Vardner, M. R. Wiesner, Modeling the concentration of volatile and semivolatile contaminants in direct contact membrane distillation (DCMD) product water. *Environ. Sci. Technol.* **51**, 13113–13121 (2017).

9. K. C. Wijekoon, F. I. Hai, J. Kang, W. E. Price, T. Y. Cath, L. D. Nghiem, Rejection and fate of trace organic compounds (TrOCs) during membrane distillation. *J. Membr. Sci.* **453**, 636–642 (2014).

10. S. Lee, A. P. Straub, Analysis of volatile and semivolatile organic compound transport in membrane distillation modules. *ACS EST Eng.* **2**, 1188–1199 (2022).

11. S. Alobaidani, E. Curcio, F. Macedonio, G. Diprofio, H. Alhinai, E. Drioli, Potential of membrane distillation in seawater desalination: Thermal efficiency, sensitivity study and cost estimation. *J. Membr. Sci.* **323**, 85–98 (2008).

12. L. W. McKeen, Fluoropolymers, in *Film Properties of Plastics and Elastomers* (Elsevier US, 2012), pp. 255–313.

13. A. Alkhudhiri, N. Darwish, N. Hilal, Membrane distillation: A comprehensive review. *Desalination* **287**, 2–18 (2012).

14. T. Merle, W. Pronk, U. von Gunten, MEMBRO$_3$X, a novel combination of a membrane contactor with advanced oxidation (O_3/H_2O_2) for simultaneous micropollutant abatement and bromate minimization. *Environ. Sci. Technol. Lett.* **4**, 180–185 (2017).

15. Y. Zhang, P. Zhao, J. Li, D. Hou, J. Wang, H. Liu, A hybrid process combining homogeneous catalytic ozonation and membrane distillation for wastewater treatment. *Chemosphere* **160**, 134–140 (2016).

16. D. L. Shaffer, J. R. Werber, H. Jaramillo, S. Lin, M. Elimelech, Forward osmosis: Where are we now? *Desalination* **356**, 271–284 (2015).

17. J. Lee, A. P. Straub, M. Elimelech, Vapor-gap membranes for highly selective osmotically driven desalination. *J. Membr. Sci.* **555**, 407–417 (2018).

18. M. Laqbaqbi, J. A. Sanmartino, M. Khayet, C. García-Payo, M. Chaouch, Fouling in membrane distillation, osmotic distillation and osmotic membrane distillation. *Appl. Sci.* **7**, 334 (2017).

19. J. Li, Y. Guan, F. Cheng, Y. Liu, Treatment of high salinity brines by direct contact membrane distillation: Effect of membrane characteristics and salinity. *Chemosphere* **140**, 143–149 (2015).

20. D. Gong, Y. Yin, H. Chen, B. Guo, P. Wu, Y. Wang, Y. Yang, Z. Li, Y. He, G. Zeng, Interfacial ions sieving for ultrafast and complete desalination through 2D nanochannel defined graphene composite membranes. *ACS Nano* **15**, 9871–9881 (2021).

21. S. Zhou, Z. Xiong, F. Liu, H. Lin, J. Wang, T. Li, H. Qiu, Q. Fang, Novel Janus membrane with unprecedented osmosis transport performance. *J. Mater. Chem. A* **7**, 632–638 (2018).

22. S. Zhou, F. Liu, J. Wang, H. Lin, Q. Han, S. Zhao, C. Y. Tang, Janus membrane with unparalleled forward osmosis performance. *Environ. Sci. Technol. Lett.* **6**, 79–85 (2019).

23. V. M. Barragán, S. Kjelstrup, Thermo-osmosis in membrane systems: A review. *J. Non-Equilib. Thermodyn.* **42**, 217–236 (2017).

24. J. G. Wijmans, R. W. Baker, The solution-diffusion model: A review. *J. Membr. Sci.* **107**, 1–21 (1995).

25. J. Lee, T. Laoui, R. Karnik, Nanofluidic transport governed by the liquid/vapour interface. *Nature Nanotechnol.* **9**, 317–323 (2014).

26. D. R. Paul, Reformulation of the solution-diffusion theory of reverse osmosis. *J. Membr. Sci.* **241**, 371–386 (2004).

27. S. Lee, A. P. Straub, Opportunities for high productivity and selectivity desalination via osmotic distillation with improved membrane design. *J. Membr. Sci.* **611**, 118309 (2020).

28. S. O. Olatunji, L. M. Camacho, Heat and mass transport in modeling membrane distillation configurations: A review. *Front. Energy Res.* **6**, 130 (2018).

29. M. S. Dariel, O. Kedem, Thermoosmosis in semipermeable membranes. *J. Phys. Chem.* **79**, 336–342 (1975).

30. S. R. D. Groot, P. Mazur, *Non-Equilibrium Thermodynamics* (Courier Corporation, 2013).

31. R. Ganti, Y. Liu, D. Frenkel, Molecular simulation of thermo-osmotic slip. *Phys. Rev. Lett.* **119**, 038002 (2017).

32. J. Kamcev, R. Sujanani, E.-S. Jang, N. Yan, N. Moe, D. R. Paul, B. D. Freeman, Salt concentration dependence of ionic conductivity in ion exchange membranes. *J. Membr. Sci.* **547**, 123–133 (2018).

33. M. S. Islam, A. Sultana, A. H. M. Saadat, M. S. Islam, M. Shammi, M. K. Uddin, Desalination technologies for developing countries: A review. *J. Sci. Res.* **10**, 77–97 (2018).

34. C. Charcosset, A review of membrane processes and renewable energies for desalination. *Desalination* **245**, 214–231 (2009).

35. S. Kjelstrup, D. Bedeaux, *Non-Equilibrium Thermodynamics Of Heterogeneous Systems* (World Scientific Publishing Company, 2008).

36. W. Chen, S. Chen, T. Liang, Q. Zhang, Z. Fan, H. Yin, K.-W. Huang, X. Zhang, Z. Lai, P. Sheng, High-flux water desalination with interfacial salt sieving effect in nanoporous carbon composite membranes. *Nature Nanotechnol.* **13**, 345–350 (2018).

37. G. Scatchard, Physical chemistry of protein solutions. I. Derivation of the equations for the osmotic pressure 1. *J. Am. Chem. Soc.* **68**, 2315–2319 (1946).

38. M. Rubinstein, *Polymer Physics* (Oxford Univ. Press, 2003).

39. D. Johnson, R. Hashaikeh, N. Hilal, Basic principles of osmosis and osmotic pressure, in *Osmosis Engineering*, N. Hilal, A. F. Ismail, M. Khayet, D. Johnson, Eds. (Elsevier, 2021), pp. 1–15.

40. J. R. McCutcheon, R. L. McGinnis, M. Elimelech, Desalination by ammonia–carbon dioxide forward osmosis: Influence of draw and feed solution concentrations on process performance. *J. Membr. Sci.* **278**, 114–123 (2006).

41. M. T. Rauter, S. K. Schnell, B. Hafskjold, S. Kjelstrup, Thermo-osmotic pressure and resistance to mass transport in a vapor-gap membrane. *Phys. Chem. Chem. Phys.* **23**, 12988–13000 (2021).

42. H. P. Hutchison, I. S. Nixon, K. G. Denbigh, The thermo-osmosis of liquids through porous materials. *Discuss. Faraday Soc.* **3**, 86–94 (1948).

43. Y.-P. Pao, Temperature and density jumps in the kinetic theory of gases and vapors. *Phys. Fluids* **14**, 1340 (1971).

44. D. Bedeaux, L. J. F. Hermans, T. Ytrehus, Slow evaporation and condensation. *Phys. A Stat. Mech. Appl.* **169**, 263–280 (1990).

45. A. P. Straub, N. Y. Yip, M. Elimelech, Raising the bar: Increased hydraulic pressure allows unprecedented high power densities in pressure-retarded osmosis. *Environ. Sci. Technol. Lett.* **1**, 55–59 (2014).

46. D. M. Davenport, A. Deshmukh, J. R. Werber, M. Elimelech, High-pressure reverse osmosis for energy-efficient hypersaline brine desalination: Current status, design considerations, and research needs. *Environ. Sci. Technol. Lett.* **5**, 467–475 (2018).

47. Q. Ge, J. Su, G. L. Amy, T.-S. Chung, Exploration of polyelectrolytes as draw solutes in forward osmosis processes. *Water Res.* **46**, 1318–1326 (2012).

48. A. B. Schantz, B. Xiong, E. Dees, D. R. Moore, X. Yang, M. Kumar, Emerging investigators series: Prospects and challenges for high-pressure reverse osmosis in minimizing concentrated waste streams. *Environ. Sci. Water Res. Technol.* **4**, 894–908 (2018).

49. S. Zhao, L. Zou, Relating solution physicochemical properties to internal concentration polarization in forward osmosis. *J. Membr. Sci.* **379**, 459–467 (2011).

50. R. Wei, S. Zhang, Y. Cui, R. C. Ong, T.-S. Chung, B. J. Helmer, J.. S. de Helmer, Highly permeable forward osmosis (FO) membranes for high osmotic pressure but viscous draw solutes. *J. Membr. Sci.* **496**, 132–141 (2015).

51. A. P. Straub, M. Elimelech, Energy efficiency and performance limiting effects in thermo-osmotic energy conversion from low-grade heat. *Environ. Sci. Technol.* **51**, 12925–12937 (2017).

52. M. Hardikar, I. Marquez, A. Achilli, Emerging investigator series: Membrane distillation and high salinity: Analysis and implications. *Environ. Sci. Water Res. Technol.* **6**, 1538–1552 (2020).

53. S. Adham, A. Hussain, J. M. Matar, R. Dores, A. Janson, Application of membrane distillation for desalting brines from thermal desalination plants. *Desalination* **314**, 101–108 (2013).

54. L. Fu, S. Merabia, L. Joly, What controls thermo-osmosis? Molecular simulations show the critical role of interfacial hydrodynamics. *Phys. Rev. Lett.* **119**, 214501 (2017).

55. J. P. G. Villaluenga, B. Seoane, V. M. Barragán, C. Ruiz-Bauzá, Thermo-osmosis of mixtures of water and methanol through a Nafion membrane. *J. Membr. Sci.* **274**, 116–122 (2006).

56. L. Song, M. Heiranian, M. Elimelech, True driving force and characteristics of water transport in osmotic membranes. *Desalination* **520**, 115360 (2021).

57. G. M. Geise, D. R. Paul, B. D. Freeman, Fundamental water and salt transport properties of polymeric materials. *Prog. Polym. Sci.* **39**, 1–42 (2014).

58. P. W. Majsztrik, M. B. Satterfield, A. B. Bocarsly, J. B. Benziger, Water sorption, desorption and transport in Nafion membranes. *J. Membr. Sci.* **301**, 93–106 (2007).

59. M. H. Cohen, D. Turnbull, Molecular transport in liquids and glasses. *J. Chem. Phys.* **31**, 1164–1169 (1959).

60. A. Tiraferri, M. Elimelech, Direct quantification of negatively charged functional groups on membrane surfaces. *J. Membr. Sci.* **389**, 499–508 (2012).

61. X. Zhou, Z. Wang, R. Epsztein, C. Zhan, W. Li, J. D. Fortner, T. A. Pham, J.-H. Kim, M. Elimelech, Intrapore energy barriers govern ion transport and selectivity of desalination membranes. *Sci. Adv.* **6**, eabd9045 (2020).

62. H. K. Lonsdale, U. Merten, R. L. Riley, Transport properties of cellulose acetate osmotic membranes. *J. Appl. Polym. Sci.* **9**, 1341–1362 (1965).

63. W. D. Mulhearn, V. P. Oleshko, C. M. Stafford, Thickness-dependent permeance of molecular layer-by-layer polyamide membranes. *J. Membr. Sci.* **618**, 118637 (2021).

64. V. K. Sharma, P. S. Singh, S. Gautam, S. Mitra, R. Mukhopadhyay, Diffusion of water in nanoporous NF polyamide membrane. *Chem. Phys. Lett.* **478**, 56–60 (2009).

65. J. Lee, A. Hill, S. Kentish, Formation of a thick aromatic polyamide membrane by interfacial polymerisation. *Sep. Purif. Technol.* **104**, 276–283 (2013).

66. J. Lee, R. Karnik, Desalination of water by vapor-phase transport through hydrophobic nanopores. *J. Appl. Phys.* **108**, 044315 (2010).

67. M. Khayet, T. Matsuura, J. I. Mengual, M. Qtaishat, Design of novel direct contact membrane distillation membranes. *Desalination* **192**, 105–111 (2006).

68. G. Vaartstra, Z. Lu, J. C. Grossman, E. N. Wang, Numerical validation of the dusty-gas model for binary diffusion in low aspect ratio capillaries. *Phys. Fluids* **33**, 121701 (2021).

69. C. K. Ho, S. W. Webb, Eds., *Gas Transport in Porous Media* (Theory and Applications of Transport in Porous Media, Springer, 2006).

70. R. Krishna, J. A. Wesselingh, The Maxwell-Stefan approach to mass transfer. *Chem. Eng. Sci.* **52**, 861–911 (1997).

71. A. Deshmukh, J. Lee, Membrane desalination performance governed by molecular reflection at the liquid-vapor interface. *Int. J. Heat Mass Transf.* **140**, 1006–1022 (2019).

72. A. Deshmukh, Understanding the impact of membrane properties and transport phenomena on the energetic performance of membrane distillation desalination. *J. Membr. Sci.*, **17**, 458–474 (2017).

73. R. R. Remick, C. J. Geankoplis, Binary diffusion of gases in capillaries in the transition region between knudsen and molecular diffusion. *Ind. Eng. Chem. Fund.* **12**, 214–220 (1973).

74. K. Malek, M.-O. Coppens, Knudsen self- and Fickian diffusion in rough nanoporous media. *J. Chem. Phys.* **119**, 2801–2811 (2003).

75. R. Marek, J. Straub, Analysis of the evaporation coefficient and the condensation coefficient of water. *Int. J. Heat Mass Transf.*, **15**, 39–53 (2001).

76. S. Garimella, T. M. Bandhauer, Measurement of Condensation Heat Transfer Coefficients in Microchannel Tubes (American Society of Mechanical Engineers Digital Collection, 2021), pp. 243–249.

77. F. Restagno, L. Bocquet, T. Biben, Metastability and nucleation in capillary condensation. *Phys. Rev. Lett.* **84**, 2433–2436 (2000).

78. B. Li, K. Bui, I. Y. Akkutlu, *Capillary Pressure in Nanopores: Deviation from Young-Laplace Equation* (Texas A&M University, 2017).

79. M. Rezaei, D. M. Warsinger, J. H. Lienhard V, M. C. Duke, T. Matsuura, W. M. Samhaber, Wetting phenomena in membrane distillation: Mechanisms, reversal, and prevention. *Water Res.* **139**, 329–352 (2018).

80. Y. Zhang, F. Shen, W. Cao, Y. Wan, Hydrophilic/hydrophobic Janus membranes with a dual-function surface coating for rapid and robust membrane distillation desalination. *Desalination* **491**, 114561 (2020).

81. Z. Wang, S. Lin, Membrane fouling and wetting in membrane distillation and their mitigation by novel membranes with special wettability. *Water Res.* **112**, 38–47 (2017).

82. O. L. I. Brown, The Clausius-Clapeyron equation. *J. Chem. Educ.* **28**, 428 (1951).

83. A. K. Ghosh, B.-H. Jeong, X. Huang, E. M. V. Hoek, Impacts of reaction and curing conditions on polyamide composite reverse osmosis membrane properties. *J. Membr. Sci.* **311**, 34–45 (2008).

84. D. Murphy, M. N. de Pinho, An ATR-FTIR study of water in cellulose acetate membranes prepared by phase inversion. *J. Membr. Sci.* **106**, 245–257 (1995).

85. H. B. Park, J. Kamcev, L. M. Robeson, M. Elimelech, B. D. Freeman, Maximizing the right stuff: The trade-off between membrane permeability and selectivity. *Science* **356**, 1137 (2017).

86. M. Ding, A. Szymczyk, A. Ghoufi, Hydration of a polyamide reverse-osmosis membrane. *J. Membr. Sci.* **501**, 248–253 (2016).

87. V. Kolev, V. Freger, Hydration, porosity and water dynamics in the polyamide layer of reverse osmosis membranes: A molecular dynamics study. *Polymer* **55**, 1420–1426 (2014).

88. D. Attarde, M. Jain, S. K. Gupta, Modeling of a forward osmosis and a pressure-retarded osmosis spiral wound module using the Spiegler-Kedem model and experimental validation. *Sep. Purif. Technol.* **164**, 182–197 (2016).

89. A. Tiraferri, A method for the simultaneous determination of transport and structural parameters of forward osmosis membranes. *J. Membr. Sci.* **16**, 523–538 (2013).

90. Y. Gao, Y.-N. Wang, W. Li, C. Y. Tang, Characterization of internal and external concentration polarizations during forward osmosis processes. *Desalination* **338**, 65–73 (2014).

91. M. Qasim, I. U. Samad, N. A. Darwish, N. Hilal, Comprehensive review of membrane design and synthesis for membrane distillation. *Desalination* **518**, 115168 (2021).

92. N. Y. Yip, M. Elimelech, Performance limiting effects in power generation from salinity gradients by pressure retarded osmosis. *Environ. Sci.* **10**, 10273–10282 (2011).

93. S. Lin, Energy efficiency of desalination: Fundamental insights from intuitive interpretation. *Environ. Sci. Technol.* **54**, 76–84 (2020).

94. S. Lin, M. Elimelech, Staged reverse osmosis operation: Configurations, energy efficiency, and application potential. *Desalination* **366**, 9–14 (2015).

95. D. Lu, Z. Zhou, Z. Wang, D. T. Ho, G. Sheng, L. Chen, Y. Zhao, X. Li, L. Cao, U. Schwingenschlögl, J. Ma, Z. Lai, An ultrahigh-flux nanoporous graphene membrane for sustainable seawater desalination using low-grade heat. *Adv. Mater.* **34**, 2109718 (2022).

96. S. Zhao, C. Jiang, J. Fan, S. Hong, P. Mei, R. Yao, Y. Liu, S. Zhang, H. Li, H. Zhang, C. Sun, Z. Guo, P. Shao, Y. Zhu, J. Zhang, L. Guo, Y. Ma, J. Zhang, X. Feng, F. Wang, H. Wu, B. Wang, Hydrophilicity gradient in covalent organic frameworks for membrane distillation. *Nat. Mater.* **20**, 1551–1558 (2021).

97. J. R. Werber, A. Deshmukh, M. Elimelech, The critical need for increased selectivity, not increased water permeability, for desalination membranes. *Environ. Sci. Technol. Lett.* **3**, 112–120 (2016).

98. J. M. Gohil, A. K. Suresh, Chlorine attack on reverse osmosis membranes: Mechanisms and mitigation strategies. *J. Membr. Sci.* **541**, 108–126 (2017).

99. R. Verbeke, V. Gómez, I. F. J. Vankelecom, Chlorine-resistance of reverse osmosis (RO) polyamide membranes. *Prog. Polym. Sci.* **72**, 1–15 (2017).

100. Z. D. Hendren, J. Brant, M. R. Wiesner, Surface modification of nanostructured ceramic membranes for direct contact membrane distillation. *J. Membr. Sci.* **331**, 1–10 (2009).

101. T. Y. Cath, V. D. Adams, A. E. Childress, Experimental study of desalination using direct contact membrane distillation: A new approach to flux enhancement. *J. Membr. Sci.* **228**, 5–16 (2004).

102. S. Lee, C. Boo, M. Elimelech, S. Hong, Comparison of fouling behavior in forward osmosis (FO) and reverse osmosis (RO). *J. Membr. Sci.* **365**, 34–39 (2010).

103. R. L. McGinnis, N. T. Hancock, M. S. Nowosielski-Slepowron, G. D. McGurgan, Pilot demonstration of the NH3/CO2 forward osmosis desalination process on high salinity brines. *Desalination* **312**, 67–74 (2013).

104. L. Fu, S. Merabia, L. Joly, Understanding fast and robust thermo-osmotic flows through carbon nanotube membranes: Thermodynamics meets hydrodynamics. *J. Phys. Chem. Lett.* **9**, 2086–2092 (2018).

105. X. Liu, L. Shu, S. Jin, A modeling investigation on the thermal effect in osmosis with gap-filled vertically aligned carbon nanotube membranes. *J. Membr. Sci.* **580**, 143–153 (2019).

106. R. M. DuChanois, C. J. Porter, C. Violet, R. Verduzco, M. Elimelech, Membrane materials for selective ion separations at the water–energy nexus. *Adv. Mater.* **33**, 2101312 (2021).

107. L. Fumagalli, A. Esfandiar, R. Fabregas, S. Hu, P. Ares, A. Janardanan, Q. Yang, B. Radha, T. Taniguchi, K. Watanabe, G. Gomila, K. S. Novoselov, A. K. Geim, Anomalously low dielectric constant of confined water. *Science* **360**, 1339–1342 (2018).

108. C. L. Ritt, J. R. Werber, M. Wang, Z. Yang, Y. Zhao, H. J. Kulik, M. Elimelech, Ionization behavior of nanoporous polyamide membranes. *Proc. Natl. Acad. Sci. U.S.A.* **117**, 30191–30200 (2020).

109. H. S. Son, Y. Kim, M. S. Nawaz, M. A. Al-Hajji, M. Abu-Ghdaib, S. Soukane, N. Ghaffour, Impact of osmotic and thermal isolation barrier on concentration and temperature polarization and energy efficiency in a novel FO-MD integrated module. *J. Membr. Sci.* **620**, 118811 (2021).

110. L. Martínez, J. M. Rodríguez-Maroto, On transport resistances in direct contact membrane distillation. *J. Membr. Sci.* **295**, 28–39 (2007).

111. S. Srisurichan, R. Jiraratananon, A. Fane, Mass transfer mechanisms and transport resistances in direct contact membrane distillation process. *J. Membr. Sci.* **277**, 186–194 (2006).

112. C.-C. Wang, On the heat transfer correlation for membrane distillation. *Energ. Conver. Manage.* **52**, 1968–1973 (2011).

113. D. M. Warsinger, S. Nejati, H. F. Juybari, H. Parmar, A. A. Alsaati, J. Swaminathan, L. M. Camacho, Performance of membrane distillation technologies, in Water Desalination: Current Status and New Developments (Y. Cohen), *The World Scientific Reference of Water Science* (Editor in-Chief, M. Tirrell) (World Scientific, 2022), vol. 3.

114. G. Wm. The Antoine equation for vapor-pressure data. *Chem. Rev.* **38**, 1–39 (1946).

115. I. Hitsov, T. Maere, K. De Sitter, C. Dotremont, I. Nopens, Modelling approaches in membrane distillation: A critical review. *Sep. Purif. Technol.* **142**, 48–64 (2015).

116. W. Sun, F. Shen, Z. Wang, Y. Zhang, Y. Wan, An ultrathin, porous and in-air hydrophilic/underwater oleophobic coating simultaneously increasing the flux and antifouling property of membrane for membrane distillation. *Desalination* **445**, 40–50 (2018).

117. N. G. P. Chew, Y. Zhang, K. Goh, J. S. Ho, R. Xu, R. Wang, Hierarchically structured Janus membrane surfaces for enhanced membrane distillation performance. *ACS Appl. Mater. Interfaces* **11**, 25524–25534 (2019).

118. L. Pisani, Simple expression for the tortuosity of porous media. *Transp. Porous Media* **88**, 193–203 (2011).
